# Seq2Saccharide: Discovering Oligosaccharides and Aminoglycosides Natural Products by Integrating Computational Mass Spectrometry and Genome Mining

**DOI:** 10.1021/jacs.5c08251

**Published:** 2025-09-18

**Authors:** Donghui Yan, Bahar Behsaz, Yanjing Li, Xiaofeng Wang, Leigh Skala, Sitong Liu, Hyun Woo Lew, Mustafa Guler, Hunsica Jayaprakash, Muqing Zhou, Liu Cao, Ashootosh Tripathi, Jason A. Clement, Taifo Mahmud, Roland D. Kersten, Hosein Mohimani

**Affiliations:** † Computational Biology Department, School of Computer Science, 6612 Carnegie Mellon University, Pittsburgh, Pennsylvania 15213, United States; ‡ Chemia Biosciences, Pittsburgh, Pennsylvania 15217, United States; § Department of Medicinal Chemistry, 1259 University of Michigan, Ann Arbor, Michigan 48109, United States; ∥ Department of Pharmaceutical Sciences, 2694 Oregon State University, Corvallis, Oregon 97331, United States; ⊥ Natural Products Discovery Core, University of Michigan, Ann Arbor, Michigan 48109, United States; # Life Sciences Institute, University of Michigan, Ann Arbor, Michigan 48109, United States; ∇ 480639 Baruch S. Blumberg Institute, 3805 Old Easton Road, Doylestown, Pennsylvania 18902, United States

## Abstract

Natural oligosaccharides and aminoglycosides are important sources of new drug candidates, especially in the development of antibiotics. In the past, discovering novel saccharides has been time-consuming and costly. However, the rapid expansion of high-throughput data, including genomic and mass spectrometry data sets, has greatly increased opportunities for natural saccharide discovery. Yet, due to the complex biosynthesis pathways of saccharides, no existing method can predict their structures with high precision. To address this, we introduce Seq2Saccharide, a tool designed to automate saccharide natural product discovery by integrating both genomic and mass spectrometry data. To enhance accuracy, Seq2Saccharide predicts hundreds or thousands of putative structures for each gene cluster. The correct structure is then identified from these predictions using a mass spectral search. Benchmarks against saccharides in the MiBIG database show that Seq2Saccharide outperforms existing methods in predicting the structure of saccharides. Furthermore, mass spectrometry analysis indicates that the variable search module can correct mispredictions from genome mining. By searching genomic and mass spectrometry data of microbial strains, Seq2Saccharide correctly identified the biosynthetic gene cluster for the polysaccharide oligosaccharide trestatin B.

## Graphical abstract

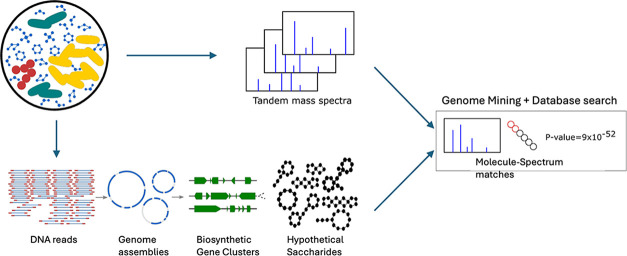

## Introduction

Natural products have an unparalleled track record in the pharmaceutical industry and continue to serve as a diverse source for discovering bioactive drug leads.^1^ Natural oligosaccharides and aminoglycosides, here referred to as saccharides, comprise a multitude of clinically approved anti-infective, anticancer, and immunosuppressive agents.^2^ Saccharides are potent antimicrobial drugs used for treating broad-spectrum infections since their first clinical use in 1944.^3^ Today, they continue to play a major role in clinical settings as antibiotics of last resort for multidrug-resistant pathogens, and many of them, e.g., amikacin (semisynthetic), gentamicin, kanamycin, neomycin, netilmicin (semisynthetic), streptomycin, and tobramycin, are routinely used as potent antibiotics.^3,4^ Moreover, several aminoglycosides and oligosaccharides, e.g., hygromycin A, spectinomycin, and avilomycin, are used frequently in veterinary and agricultural applications.^4–6^

The last seven decades of effort in saccharide discovery largely focused on the most abundant bioactive compounds from microbial isolates. However, this approach is time- and labor-intensive and leads to the frequent rediscovery of already-known compounds. At the same time, pathogens have gained resistance against the last-resort antibiotics, emphasizing the urgent need for new antibiotic drugs.

In recent years, advances in large-scale omics technologies have enabled the acquisition of untargeted tandem mass spectrometry (metabolomics) and genomic sequencing data from tens of thousands of microbial isolates and environmental/host-oriented microbial communities.^7–10^ However, genome mining studies of data from the National Center for Biotechnology Information (NCBI) and the Joint Genome Institute (JGI) repositories have revealed hundreds of orphan biosynthetic gene clusters (BGC, genes that code for the assembly and tailoring of natural products) that make/synthesize with still uncharacterized small molecules. For example, the Integrated Microbial Genome Atlas of Biosynthetic Gene Clusters (IMG-ABC)^11^ reported over 500,000 BGCs encoding small molecules in public genome assemblies, but only 640 of them have been connected to their molecular products.^11,12^ This small rate signifies a gap between the limited number of BGCs with characterized natural products and the massive number of sequenced BGCs. Among uncharacterized microbial BGCs, saccharide-encoding pathways have been predicted to represent a large fraction of uncharacterized biosynthetic genes in microbial genomes.^13^

Alongside the experimental advances, computational methods for natural product identification have been developed to analyze either genomic data^14,15^ or metabolomics data.^16–19^ Moreover, scalable computational methods integrating unannotated mass spectra with uncharacterized BGCs to automate the discovery of peptidic natural products have been developed and applied to large-scale data sets, leading to the discovery of new families of ribosomally synthesized and post-translationally modified peptides (RiPP),^20–22^ polyketides,^23^ and nonribosomal peptides.^24,25^ These methods demonstrate the power of integrative analysis of metabolomics and genomics data. Currently, however, no efficient methods exist for integrating genomics and metabolomics to discover oligosaccharides or aminoglycosides.

Large-scale analysis of saccharide natural products is a particularly challenging task due to their complex biosynthesis and chemistry. Oligosaccharides are carbohydrates made up of chains of monosaccharide building blocks, and their biosynthesis is facilitated by enzymes encoded by genomic sequences. In addition to amino-sugar monomers, aminoglycosides contain an aminocyclitol ring, connected by glycosidic bonds.^4,26^ Like other classes of oligomeric natural products, saccharides are synthesized through sequences of condensation and modification reactions, facilitated by enzymes encoded by genomic sequences, to create core backbone scaffolds. Similar to noncolinear nonribosomal peptides and polyketides, the number and order of the monomers in saccharide backbones is not strictly determined by the order of enzyme-encoding genes in the BGC locus. In addition to the enzyme-enabled monomers, many saccharides include enzyme-independent metabolites in their backbone scaffolds, such as glucosamine and ribose, which cannot be predicted from the BGC sequences.^27^ Due to this, every possibility of backbone structure/organization is feasible for each saccharide BGC, resulting in extraordinarily large numbers of potential structures. Moreover, these core backbones often undergo further postassembly modifications such as oxidation, methylation, glycosylation, etc.^28^ This intricate biosynthesis process in saccharides complicates the automated prediction of natural saccharides from microbial genomic data.^14^ Currently, only two automated genome-mining-based prediction methods are available for saccharide BGCs, namely PRISM^14^ and antiSMASH.^15^ However, our benchmarks show that these methods can not correctly identify the product of any of the saccharide BGCs in the MIBiG benchmark data set^12^ using a Tanimoto similarity threshold of 0.95 and only one BGC has Tanimoto similarity over 0.85. The Tanimoto similarity threshold is a metric used to quantify the structural similarity between two chemical compounds, calculated as the ratio of shared molecular features to the total number of features present in either compound.^29,30^ Furthermore, these methods do not support large-scale mass spectrometry analyses for the scalable discovery of saccharides.

To address current limitations, we developed Seq2Saccharide to aid in the discovery of aminoglycoside and oligosaccharide natural products. Seq2Saccharide is the first automated pipeline that integrates microbial BGCs with tandem mass spectrometry data for the identification of saccharide natural products. Seq2Saccharide employs a probabilistic model to predict monomers that are encoded in the BGC, and uses a rule-based approach to further predict monomers that are not encoded in the BGC (primary monomers). These monomers are then assembled in the predicted sequence to construct the initial backbone. Then, accessory enzymes facilitate postassembly modifications to produce the mature structures. Finally, mass spectra are compared against the predicted molecular structures using an error-tolerant database search, and matches with high scores and low p-values are reported. By applying Seq2Saccharide to a data set of *Streptomyces* strains, Seq2Saccharide correctly identified known biosynthetic pathways for streptomycin, neomycin, paromomycin, kanamycin, and ribostamycin without any prior knowledge regarding biosynthetic pathway. Additionally, Seq2Saccharide correctly identified the biosynthetic gene cluster of trestatin B from *Streptomyces ansochromogenes* subsp. *pallens* NRRL B-12018.

## Results

### Biosynthetic Pathway for Saccharides

The formation of a saccharide compound is a complex process that involves three major steps: sugar monomer creation, bond formation, and postassembly modifications (Figure 1). Seq2Saccharide customizes different approaches to account for each of these steps and construct hypothetical saccharide structures (Figure 1).

**Figure 1:**
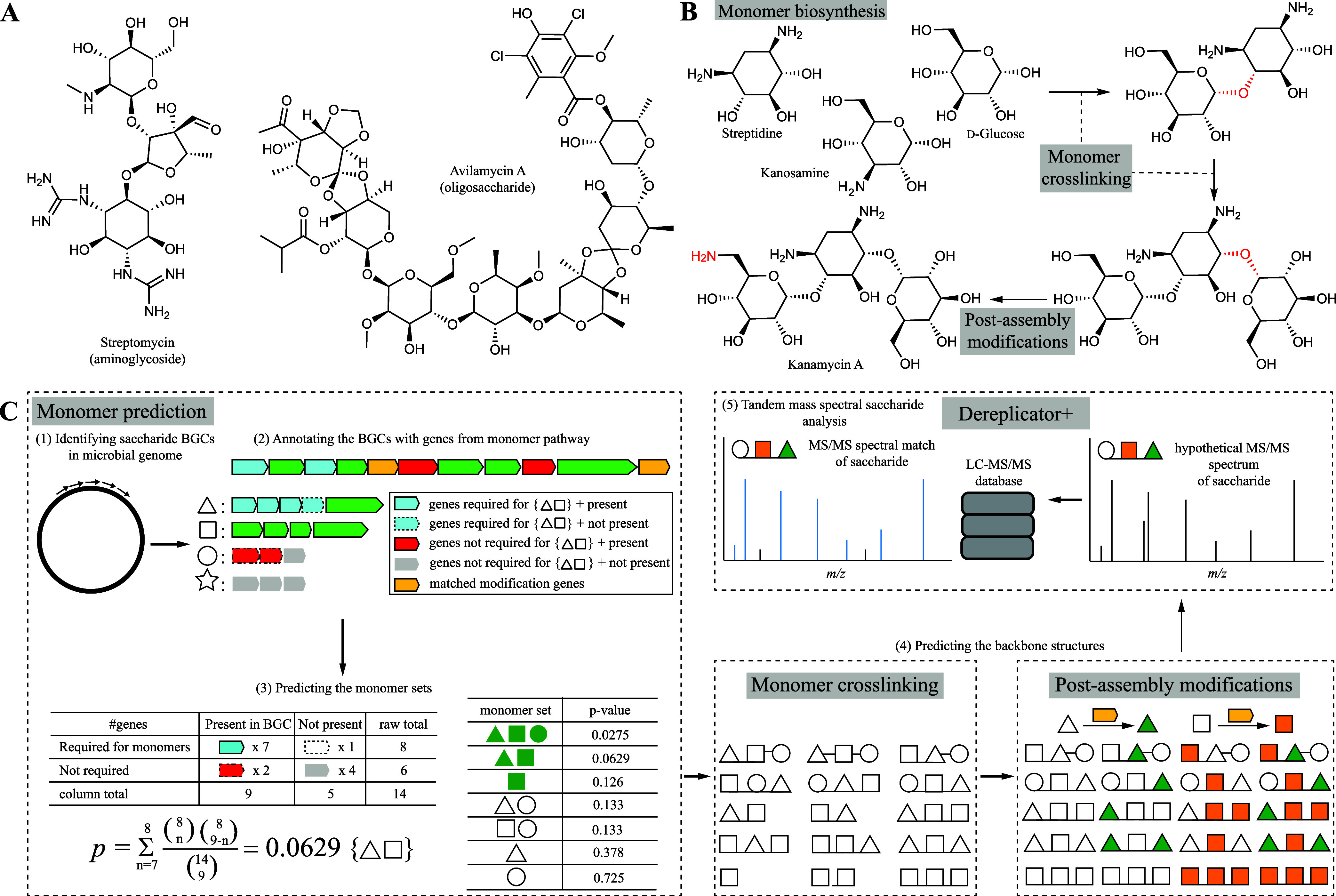
Seq2Saccharide pipeline for predicting the structure of candidate saccharides from their biosynthetic gene clusters. (A) Representative aminoglycoside and oligosaccharide structures targeted by Seq2Saccharide. (B) Biosynthetic logic of saccharide natural products targeted by Seq2Saccharide exemplified by kanamycin A biosynthesis.^31^ (C) Seq2Saccharide pipeline. During monomer prediction: (1) DNA sequences are mined to identify saccharide-encoding BGCs. A database of genes required for the biosynthesis of each monomer is then created, with each monomer represented by a distinct shape (triangle, square, circle, or star). Examples of these monomers are illustrated in [Fig fig1](B) as streptidine, kanosamine, and glucose. (2) Tailoring modification enzymes are identified via HMM search, and a list of genes required for the biosynthesis of each monomer is compiled, as the colored genes shown. For each BGC, various combinations of monomers (monomer sets) are considered. (3) The likelihood of each monomer set is determined based on the overlap between required genes and those present in the BGC. Note that a single gene can contribute to the biosynthesis of multiple monomers. A statistical significance score is assigned to each monomer set and its corresponding BGC by the probabilistic model. (4) The most likely monomer sets are connected in the predicted order to form candidate backbones, with postassembly modifications incorporated, as the step shown in Figure 2. (5) Final mature structures are matched against mass spectra using Dereplicator+.^17^

Saccharide formation begins with the production of sugar monomers derived from central metabolic intermediates such as glucose-6-phosphate, fructose-6-phosphate, or ribulose-5-phosphate. These intermediates are enzymatically transformed into activated sugar nucleotides, such as UDP-glucose, GDP-mannose, or CMP-sialic acid, by enzymes encoded within the saccharide biosynthetic gene clusters (BGCs). These enzymes work together to generate sugar monomers essential for saccharide construction in subsequent steps by providing the energy required for glycosidic bond formation. Seq2Saccharide employs an HMM-based search algorithm to identify the function of each gene in the BGC, and the enzymes these annotated genes represent are then fed into a probabilistic model to predict the resulting sugar monomers.

The next step involves assembling the individual sugar monomers into a saccharide backbone. Glycosyltransferases catalyze this process by sequentially adding sugar monomers to an acceptor molecule, forming glycosidic bonds with high specificity. These enzymes determine the type of linkage and the branching structure of the saccharide. Typically, the bond formed between two sugar monomers remains consistent across different saccharide compounds (Figure 2). Seq2Saccharide utilizes a rule-based approach to catalog all possible bond types and applies the appropriate bonds during monomer assembly to construct the saccharide backbone.

**Figure 2:**
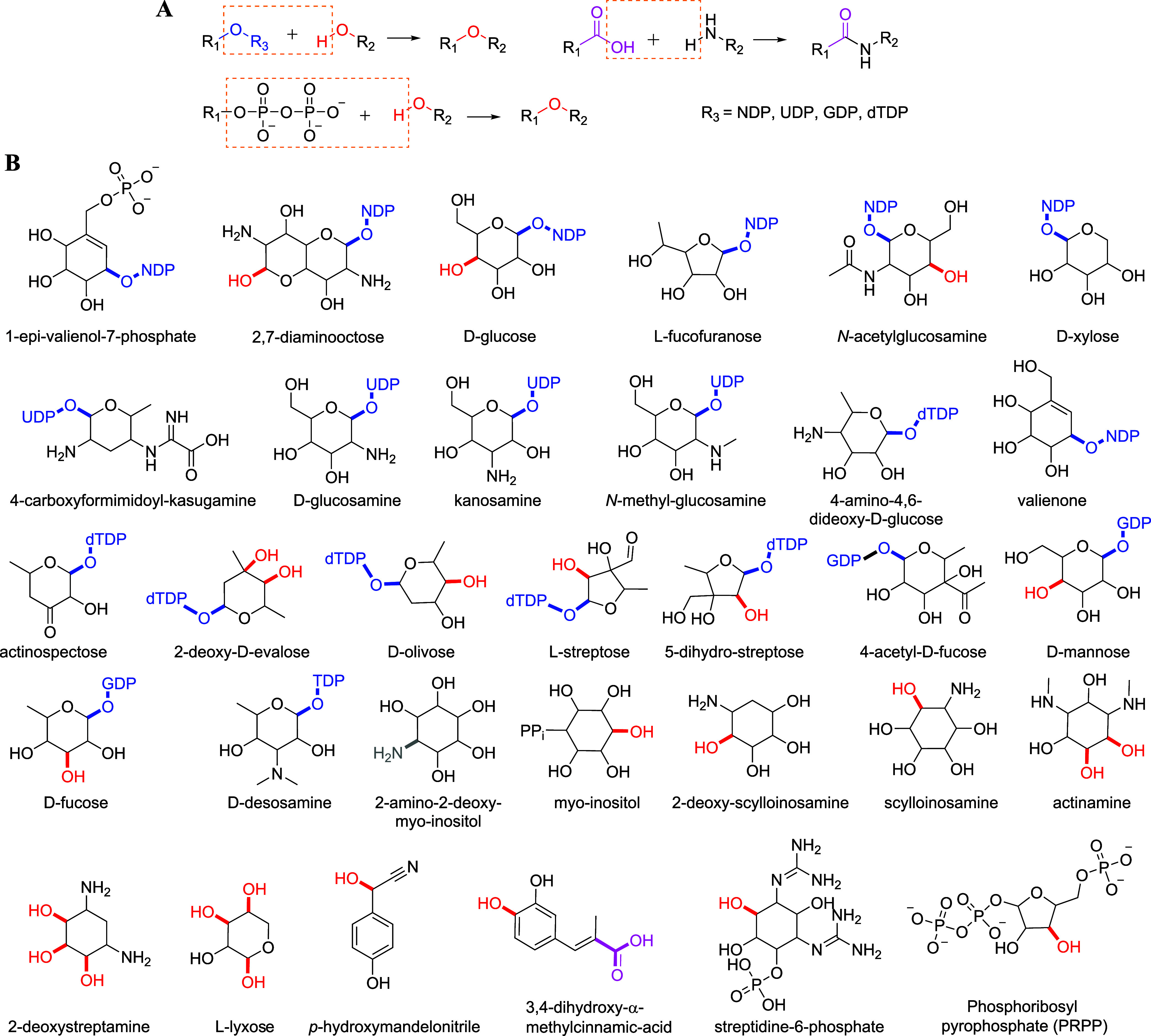
(A) Bond formation rules between saccharide monomers (monomer cross-linking step), where in the first reaction, the R3 group represents NDP (nucleoside diphosphate), GDP (guanosine diphosphate), UDP (uridine diphosphate), dTDP/TDP (thymidine diphosphate). (B) 2D structures of saccharide monomers predicted by Seq2Saccharide, with their reactive groups highlighted in blue, red, brown, green, and pink. Seq2Saccharide predicts 2D structures given the achiral nature of tandem mass spectrometry data.

Once the saccharide backbone is formed, postassembly modification enzymes tailor the structure to enhance its biological functions and stabilize the molecule. These modifications include methylation by *O*-methyltransferases, acetylation by acetyltransferases, sulfation by sulfotransferases, and oxidation by oxidoreductases (Figure 3). For example, oxidation of sugar hydroxyl groups to carboxyl groups enhances hydrophilicity, whereas methylation increases hydrophobicity.^32^ Such modifications influence saccharide bioactivity and stability, making them suitable for diverse roles, including cell signaling,^33^ structural integrity,^34^ or antibiotics.^6^ Seq2Saccharide incorporates a postassembly modification database that links specific genes to corresponding modifications. Once these modified genes are identified in the genome, a graph-based approach is used to apply the modifications to the backbone, forming mature compounds automatically. An example is the biosynthesis of kanamycin A in which a postassembly amination can occur on the intermediate saccharide 2-deamino-2′-hydroxyparomamine before final monomer cross-linking with kanosamine to yield kanamycin A.^35^

**Figure 3:**
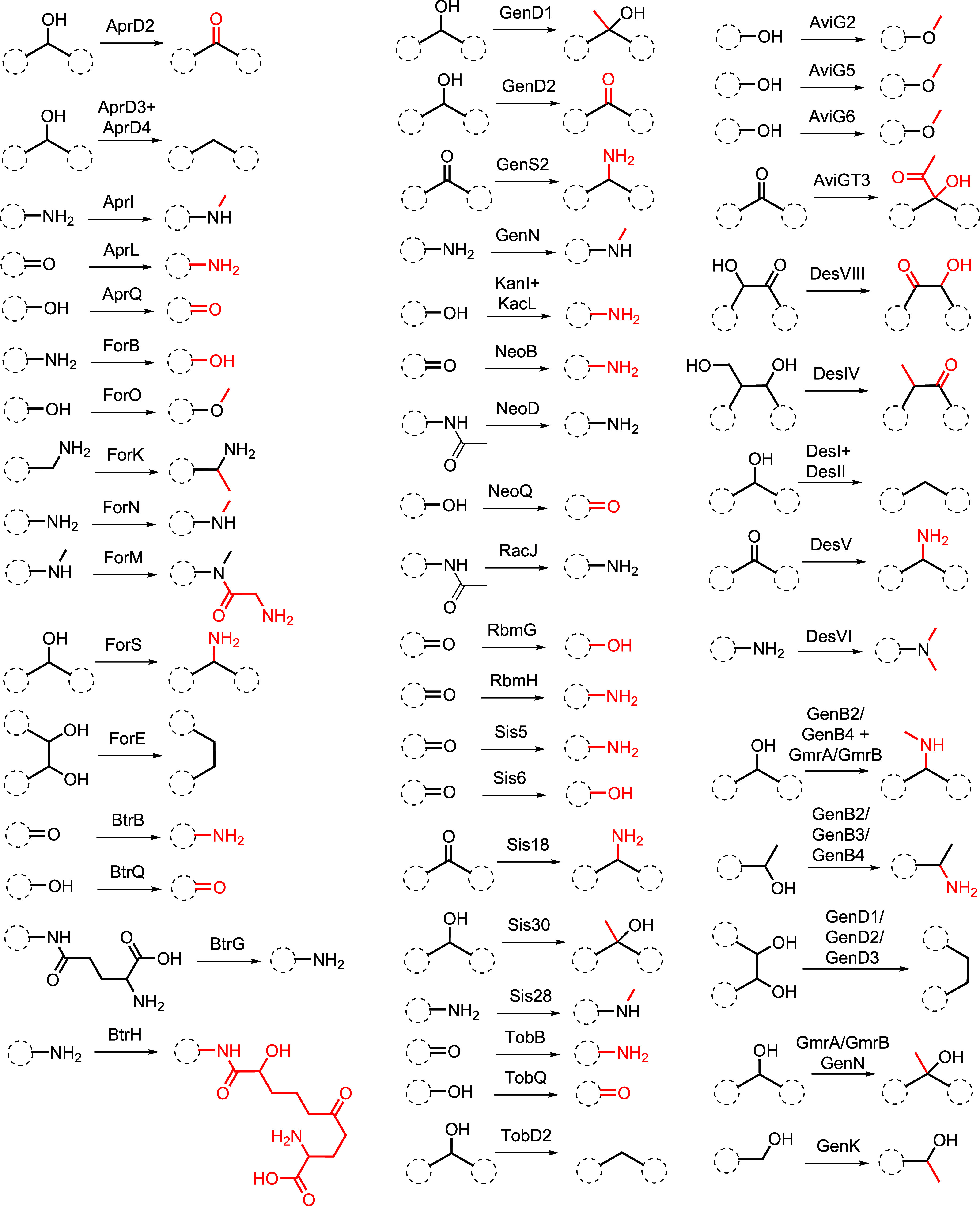
List of 49 oligosaccharides postassembly modifications gathered through literature survey. Modification sites and reactions are highlighted in red. Enzymes responsible for each modification are also shown. Modifications requiring more than one enzyme are noted with a plus sign between the enzymes.

Seq2Saccharide integrates these processes into an automated genome mining pipeline, providing a comprehensive tool for predicting saccharide structures from BGCs in microbial genomes, generating tandem mass spectra of the predicted saccharide structures and matching them to tandem mass spectra in matching metabolomic data for the efficient discovery of saccharides from a genome-sequenced bacterium (Figure 1).

### Seq2Saccharide Overview

Figure 1C illustrates the Seq2Saccharide pipeline, which includes the following steps as described in the methods section: (1) DNA sequences are mined to identify saccharide-encoding BGCs. A database of genes required for the biosynthesis of each monomer is then created, with each monomer represented by a distinct shape (triangle, square, circle, or star). (2) Tailoring modification enzymes are identified via HMM search, and a list of genes required for the biosynthesis of each monomer is compiled, as the colored genes shown. (3) The likelihood of each monomer set is determined based on the overlap between required genes and those present in the BGC. (4) The most likely monomer sets are connected in the predicted order to form candidate backbones, with postassembly modifications incorporated. (5) Final mature structures are matched against mass spectra using Dereplicator+.^17^ Figure 4 provides a detailed representation of the biosynthetic pathway of Streptomycin as a real-world example.

**Figure 4:**
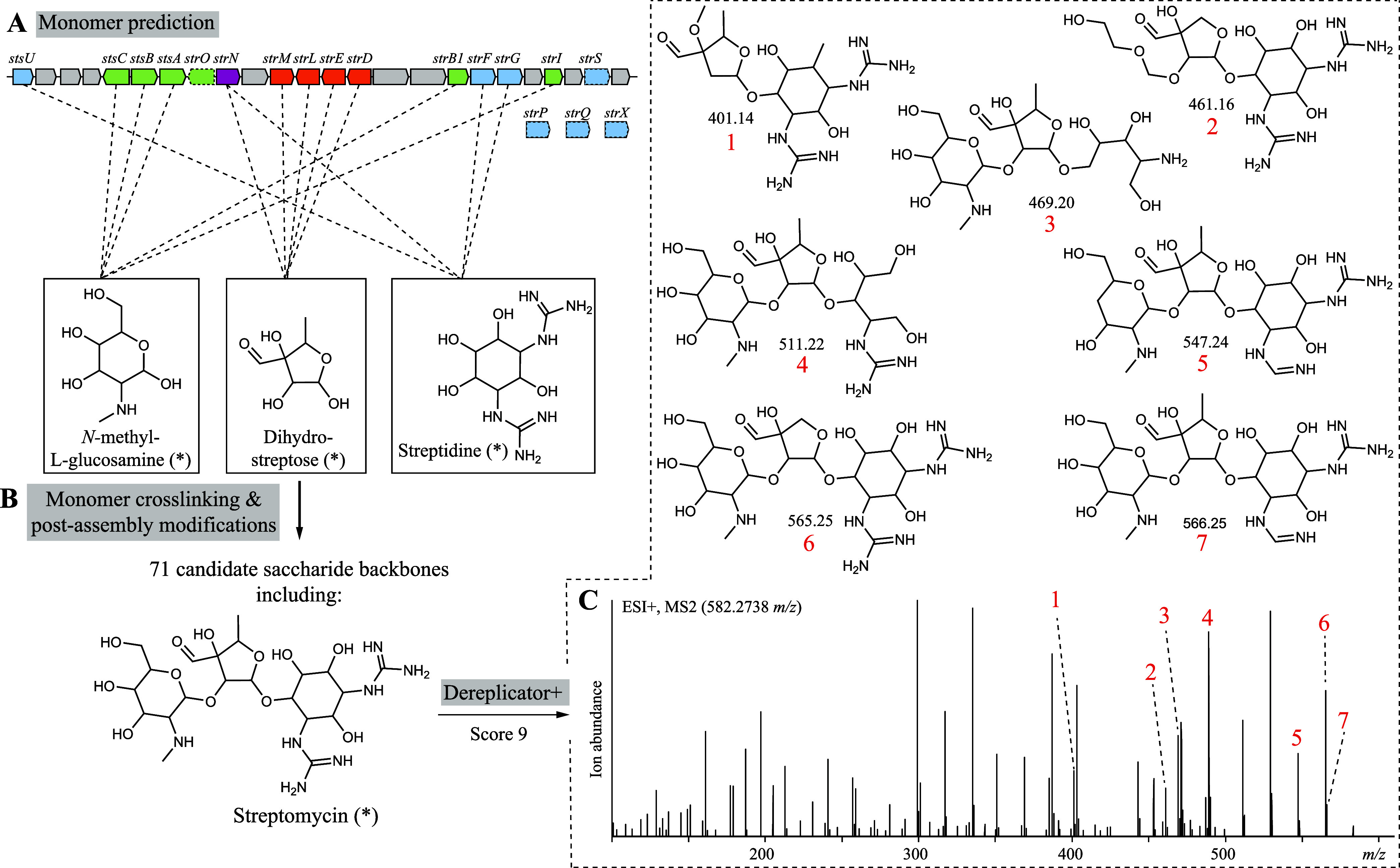
Identification of streptomycin using Seq2Saccharide in *Streptomyces* sp. ISP-5236. (A) Gene annotation results for streptomycin. Genes annotated in the genome are color-coded to indicate their roles: green for, orange for dihydrostreptose, and blue for streptidine. Seq2Saccharide identified five potential genes involved in the formation of streptidine (shown in blue), four potential genes involved in the formation of dihydrostreptose (shown in orange), and three potential genes involved in the formation of *N*-methyl-l-glucosamine (shown in green). The purple-colored gene (*strN*) participates in the biosynthesis of both dihydrostreptose and streptidine. Fully colored genes represent successful detection via HMM search, while dashed outlines indicate genes (*strO* and *strS*) that the HMM search failed to identify. The second row shows essential genes (*strP, strQ, strX*) located outside the BGC, not captured by the algorithm. (B) Seq2Saccharide prediction of saccharide structures from identified monomers. Seq2Saccharide predicts 2D structures (denoted by *) due to the achiral nature of general tandem mass spectrometry data. Subsequent cyclization leads to the formation of streptomycin (according to the figure). (C) Dereplicator+ matching of streptomycin structure predicted from the streptomycin gene cluster with tandem mass spectra from a *Streptomyces* extract LC-MS/MS data set. Fragments from streptomycin and corresponding peaks in the tandem mass spectrum are highlighted in blue.

### Data Sets

Seq2Saccharide was benchmarked using a database of 20 known oligosaccharides/aminoglycosides and their associated BGCs from MIBiG.^12,31,36–50^ Eighteen of these compounds are pure saccharides, not containing structures from any other natural product classes, whereas avilamycin is an oligosaccharide-polyketide hybrid and dhurrin is a cyanogenic glycoside.^36,50^ Nineteen of 20 test compounds are microbial natural products, whereas dhurrin is a plant natural product. Their biosynthetic information was obtained through a literature search.

### Benchmarking Gene Annotation Accuracies

Table 1 presents the accuracy of predictions for genes responsible for monomer synthesis and enzymes responsible for postassembly modifications in 20 saccharide BGCs from MIBiG. A comprehensive literature survey identified genes reported as essential for the synthesis of each monomer and for postassembly modifications. In 7 of the 20 BGCs, all previously reported synthesis genes were identified within these BGCs. Similarly, for 15 of the BGCs, all previously reported modification enzymes were present. On average, 77.17% of the synthesis genes and 79.17% of the modification enzymes previously reported were detected. Some essential genes and enzymes are located outside the BGC within the producing organism’s genome (Figure 4A). Such occurrences are rare, and currently it is challenging to handle these genes due to increase in computational cost and false discovery rate. Seq2Saccharide mitigates this issue using a probabilistic model to predict the most likely monomers, even when some genes are unannotated.

**Table 1:** Seq2Saccharide Prediction Accuracy of Biosynthesis and Modification Genes^a^

BGC ID (MIBiG/NCBI)	molecule name	#required genes	present genes (%)	#modification genes	present modification genes (%)
BGC0000026	avilamycin	9	100% (9)	5	100% (5)
BGC0000689	2-deoxystreptamine	4	75% (3)	0	–
Y18523.4	acarbose	11	72.7% (8)	0	–
BGC0000692	apramycin	10	80% (8)	6	83.3% (5)
BGC0000693	butirosin	4	75% (3)	4	100% (4)
AJ628421.2	fortimicin	1	0% (0)	7	85.7% (6)
BGC0000696	gentamicin	4	50% (2)	7	0% (0)
BGC0000698	hygromycin A	14	92.9% (13)	0	–
BGC0000700	istamycin	2	100% (2)	1	0% (0)
BGC0000702	kanamycin	5	100% (5)	5	100% (5)
BGC0000708	lividomycin	4	100% (4)	0	–
BGC0000707	kasugamycin	3	100% (3)	0	–
BGC0000711	neomycin	4	75% (3)	2	100% (2)
BGC0000712	paromomycin	4	75% (3)	0	–
BGC0000713	ribostamycin	4	75% (3)	3	100% (3)
BGC0000714	sisomicin	4	50% (2)	5	100% (5)
BGC0000716	spectinomycin	13	100% (13)	0	–
BGC0000719	tobramycin	5	100% (5)	3	100% (3)
BGC0000724	streptomycin	18	72.2% (13)	0	–
BGC0000798	dhurrin	2	50% (1)	0	–

a For monomers and modifications in each saccharide from MIBiG, we conducted a literature survey to identify all biosynthesis genes and modification enzymes that are reported to be required. We then computed the ratio and number of such required genes and enzymes identified by HMM search in the BGC.

### Benchmarking Saccharide Backbone Construction and Postassembly Modification Modules

Seq2Saccharide employs a probabilistic model to identify the set of monomers present in a saccharide, based on the genes present in the BGC. After identifying all the monomer synthesis genes in the BGC, Seq2Saccharide generates all potential sets of monomers. These sets are then ranked based on *p*-values derived from the probabilistic model, with the top-ranked sets assembled into candidate backbone structures. Monomer sets that cannot be assembled due to noncompatible bond formation rules are discarded. Note that in the probabilistic model, some monomers can still be included in the sets even if not all the genes responsible for their recruitment are identified by the HMM search.

After the main saccharide structure (backbone) is assembled, it undergoes additional chemical modifications that are essential for its stability and bioactivity. These modifications are performed by postassembly enzymes, which add or alter chemical groups (like methyl or acetyl groups) on the backbone. The presence of these enzymes is predicted by analyzing specific genes in the organism’s biosynthetic gene cluster (BGC), a collection of genes that work together to produce complex molecules. By identifying these genes, we can better predict the chemical modifications that are applied to the saccharide backbone to yield mature saccharide compound.

To facilitate this, we mined the literature to construct a database of saccharide postassembly enzymes and their corresponding modifications. The database was parsed into a computer-readable format, featuring a substrate motif (stored as a SMILES string) along with a series of graph modifications (involving the addition or removal of nodes and edges). These modifications are applied when the molecular structures match the substrate motifs.

For 12 BGCs sourced from the MIBiG database, Seq2Saccharide accurately reconstructs their molecular structures starting from the genome sequence (Table 2). In addition to the monomers for which the synthesis genes are encoded in the BGC, Seq2Saccharide also considers up to two primary monomers (d-mannose,^51^ d-glucose,^52^ d-glucosamine,^53^ *N*-acetylglucosamine,^54^ d-xylose,^51^ and d-ribose^55^), which are not encoded in the BGC. While this significantly improves the prediction accuracy of molecular products, it increases the number of candidate structures predicted by Seq2Saccharide.

**Table 2:** Seq2Saccharide Structure Prediction from Genome Sequence^a^

molecule name	#monomers	#primary monomers	correct monomer set rank	#backbones	best tanimoto similarity
avilamycin	7	2	N/A	240	0.42
2-deoxystreptamine	1	0	330/14,014	649	1
acarbose	4	1	195/277,530	69	1
apramycin	3	1	35/52,338	748	0.46
butirosin	3	2	121/1364	758	0.7125
fortimicin	2	1	4/14,014	648	0.3
gentamicin	3	2	165/14,014	649	0.36
hygromycin	3	0	N/A	445	0.38
istamycin	2	1	15/8382	648	0.45
kanamycin	3	1	80/14,014	626	1
lividomycin	5	2	106/22,506	747	1
kasugamycin	2	1	6/76,384	64	1
neomycin	4	2	106/8382	626	1
paromomycin	4	2	218/34,870	529	1
ribostamycin	3	2	106/22,506	634	1
sisomicin	3	2	135/34,870	649	0.48
spectinomycin	2	0	40/366,586	52	1
tobramycin	3	1	80/14,014	626	1
streptomycin	3	0	70/9401	71	1
dhurrin	2	1	2/22	26	1

a The table shows the number of monomers, primary monomers, the rank of the correct monomer set, the number of candidate backbone structures, and the highest Tanimoto similarity to the correct structure among all candidate structures. The top 500 ranked monomer sets are selected to generate candidate backbones.

### Benchmarking Saccharide Structure Prediction

Existing tools like antiSMASH^15^ and Gecco^56^ focus on identifying BGCs and ORFs responsible for saccharide compound production but are unable to predict the final compounds these BGCs generate. PRISM,^14^ although capable of predicting final compounds, is optimized for polyketides and NRPs rather than saccharides. It also fails to account for postassembly modifications or use mass spectrometry data to filter candidates. In contrast, Seq2Saccharide predicts the final saccharide product from identified BGCs and refines predictions with mass spectrometry data, providing more reliable structures.

To demonstrate Seq2Saccharide’s capabilities, we analyzed 20 saccharide BGCs from the MIBiG database^12^ (Figure 5). Seq2Saccharide successfully identified 12 known saccharides without prior knowledge regarding the biosynthetic pathway, whereas PRISM^14^ failed to identify any. Among PRISM’s predictions, only four molecules had a Tanimoto similarity above 0.7.

**Figure 5:**
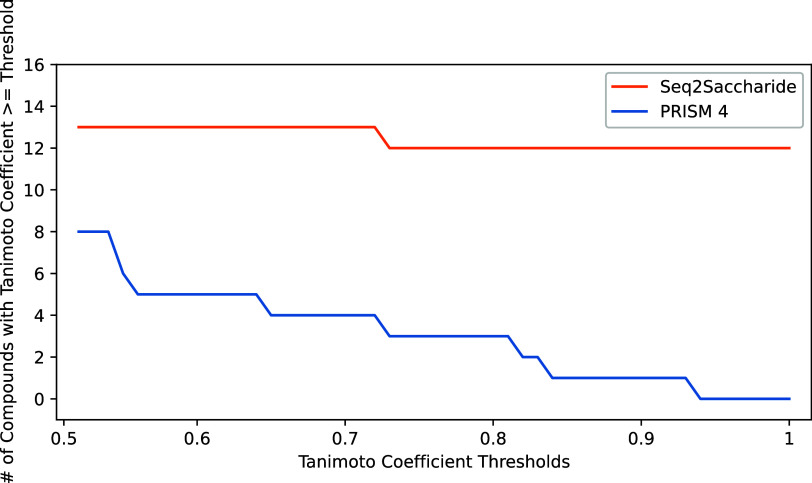
Number of correctly predicted saccharides by Seq2Saccharide and PRISM^14^ at different Tanimoto similarity thresholds. The benchmark data set comprises 20 known saccharide BGCs from the MIBiG database. The Tanimoto similarity for Seq2Saccharide is calculated by running the software on the genome sequence of producers, predicting the hypothetical structure, and comparing it to the true structure. In contrast, the Tanimoto similarity for PRISM is taken directly from Skinnider et al.^14^

### Applying Seq2Saccharide to Large-Scale Mass Spectrometry and Genomic Data Sets

The Global Natural Products Social (GNPS) Molecular Networking platform is an open-access resource for storing, analyzing, sharing, and visualizing mass spectrometry data.^10^ We generated candidate molecules for saccharide BGCs from *Streptomyces* and searched them against *Streptomyces* mass spectral data from GNPS data sets using Variable Dereplicator+.^17^ Variable Dereplicator+ is an enhanced version of Dereplicator+ that accommodates the absence of certain monomers/modifications by incorporating unknown mass shifts during the matching process. After searching the candidate structures against *Streptomyces* data sets from GNPS, we identified five known saccharides at a p-value threshold of 10^–3^. These molecules include streptomycin from *Streptomyces* sp. ISP-5236 (Figure 4), neomycin B from *Streptomyces albogriseolus* ISP-5003 (Supporting Figure 1), paromomycin from *Streptomyces catenulae* ISP-5258 (Supporting Figure 2), kanamycin A from *Streptomyces kanamyceticus* ISP-5500 (Supporting Figure 3), and ribostamycin from *Streptomyces ribosidificus* ATCC 21294 (Supporting Figure 4).

### Identification of Streptomycin

After analyzing the genome sequence of *Streptomyces* sp. ISP-5236, Seq2Saccharide identified five potential genes involved in the formation of streptidine, four potential genes for dihydrostreptose, and three potential genes for *N*-methyl-l-glucosamine. The correct monomer set streptidine, dihydrostreptose, and *N*-methyl-l-glucosamine was ranked 70th among 9401 candidate monomer sets. The top 500 ranked monomer sets were selected, and among them, 71 sets resulted in valid backbones after the application of bond-formation rules. Feasible modifications were applied to each candidate’s backbones. The masses and structures of these mature candidates (in SMILES format) were stored in a comma-separated file for searching against a mass spectral data set.

Then, Dereplicator+ generated the theoretical mass spectrum for each candidate structure,^16,17^ compared them with experimental spectra, generated a score for each candidate structure and spectrum pair, and assigned a *p*-value to each pair. The scoring match (score 7, *p*-value 1.29 × 10^–4^) corresponded to the correct structure of streptomycin (Figure 4).

### Identification of a Putative BGC for Trestatin B

We wanted to test if Seq2Saccharide could identify new saccharide natural products and their BGCs from large-scale omics data. Seq2Saccharide was applied to search paired omics data sets and it identified two candidate oligosaccharide BGCs with matched saccharide analytes in metabolomic data sets from the genome sequence of *S. ansochromogenes* subsp. *pallens* NRRL B-12018 (Figure 6) and *Streptomyces albofaciens* ATCC 23783 (Supporting Figure 5). Each saccharide BGC was translated by Seq2Saccharide into 71 candidate oligosaccharide backbones (Figure 6B and Supporting Figure 5). A Variable Dereplicator+ search of these structures against the corresponding spectra led to the identification of a high-scoring molecule-spectrum match for these saccharide BGCs with a matching score of 45 and a *p*-value of 1.8 × 10^–4^. Searching the high-scoring candidate structures against the PubChem database^57^ identified them as α-amylase inhibitors trestatin B and acarviostatin I03 (Figure 6B,C and Supporting Figure 5). Candidate biosynthetic gene clusters for acarviostatin I03 and trestatin B have been reported from *S. coelicoflavus* *ZG0656* and *Streptomyces dimorphogenes* ATCC 3148, respectively.^58,61^ The putative trestatin BGC identified by Seq2Saccharide from *S. ansochromogenes* subsp. *pallens* NRRL B-12018 contains biosynthetic genes for the production of valienone and 4-amino-4,6-dideoxy-d-glucose monomers from trestatin B (Figure 6A and Supporting Table 1).^59^ The identified saccharide BGC from *S. albofaciens* ATCC 23783 showed high homology to the putative acarviostatin BGC from *S. coelicoflavus* ZG0656 (Supporting Table 2). Acarviostatin I03 and trestatin B are both oligosaccharides which contain an acarviosine core structure of valienamine cross-linked to a 4-amino-4,6-dideoxy-d-glucose. The 4-amino-4,6-dideoxy-d-glucose is linked to three d-glucose monomers via α-1,4-glycosidic bonds. Trestatin B and acarviostatin I03 differ in the glycosidic bond of the terminal disaccharide unit which is connected via an α,α-1,1-bond in trestatin B and an α-1,4-linkage in acarviostatin I03 (Figure 7A and Supporting Figure 6).

**Figure 6:**
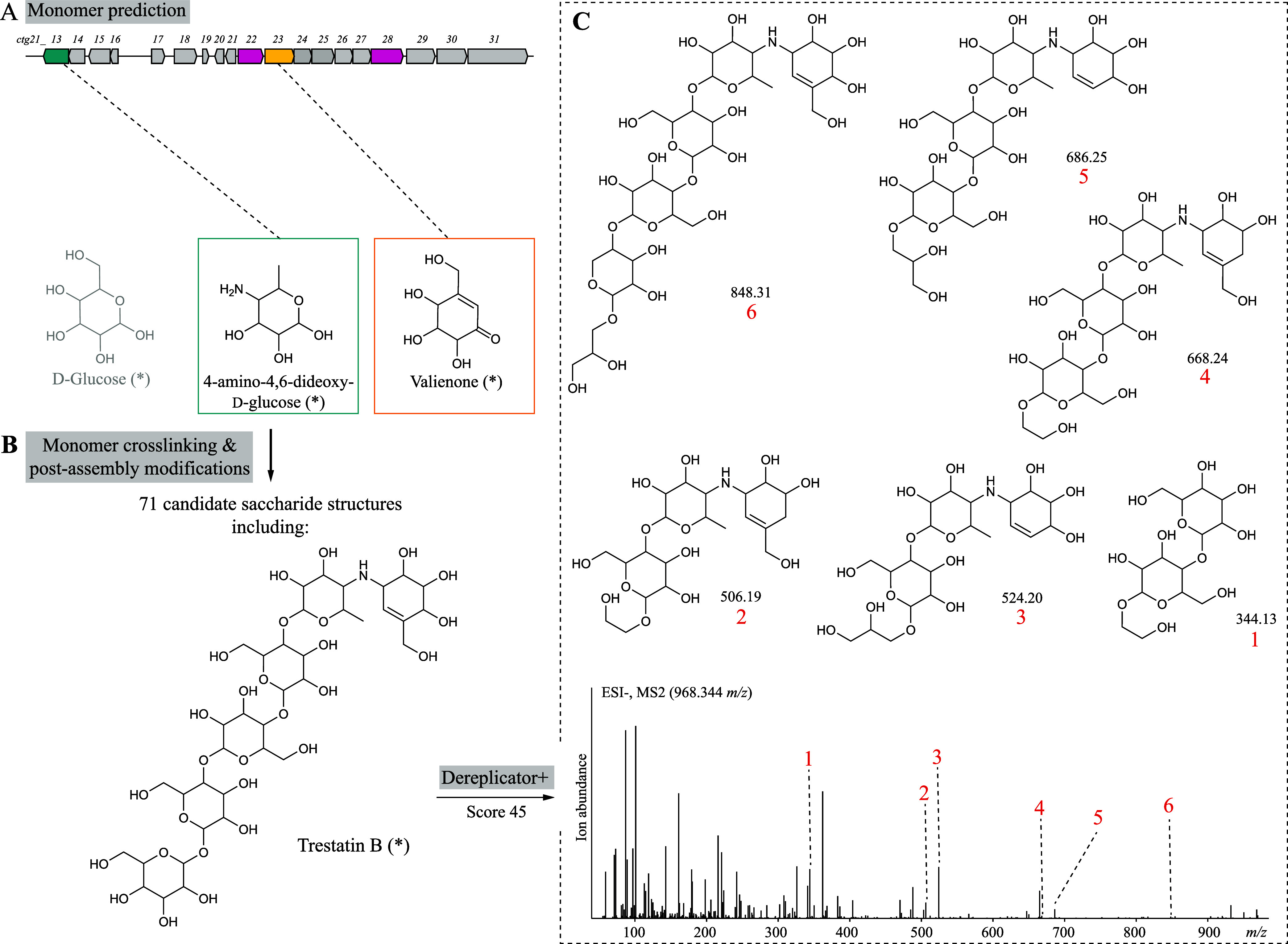
Identification of trestatin B using Seq2Saccharide. Seq2Saccharide predicts 2D structures given the achiral nature of general tandem mass spectrometry data (denoted by *). (A) Seq2Saccharide identified one potential gene involved in the formation of valienone (shown in orange) and one potential gene for 4-amino-4,6-dideoxy-d-glucose (shown in green). An additional monomer, glucose, is included as a primary monomer without corresponding genes in the search (shown in gray). Subsequent cyclization led to the formation of trestatin B. (B) Trestatin B structure predicted by Seq2Saccharide from target saccharide BGC in *S. ansochromogenes* subsp. *pallens* NRRL B-12018. (C) Dereplicator+ identification of fragments from trestatin B in a tandem mass spectrum of *S. ansochromogenes* subsp. *pallens* NRRL B-12018 extract.

**Figure 7:**
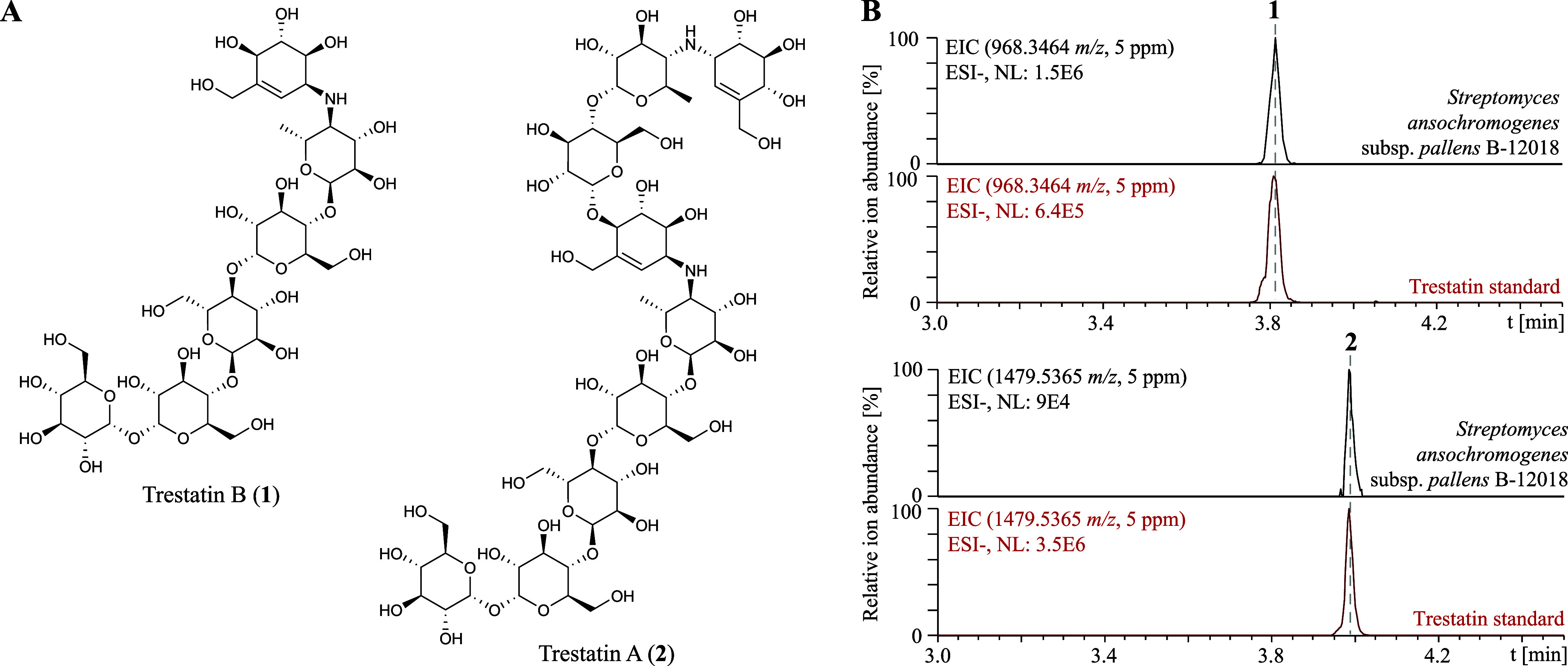
Trestatin characterization in *S. ansochromogenes* subsp. *pallens* B-12018. (A) Structures of trestatins A and B. (B) Base peak chromatogram of trestatin B acquired in negative ion mode from the culture supernatant of *S. ansochromogenes* subsp. *pallens* B-12018. Tandem mass spectrum of trestatin B detected in the culture supernatant of *S. ansochromogenes* subsp. *pallens* B-12018, with annotated fragment ions corresponding to putative trestatin B substructures. Trestatin B was detected as a [M + NH_3_]^−^ analyte. The trestatin standard contains trestatins A and B (Supporting Figure 7).

### Experimental Validation of the Trestatin B BGC

To verify the Seq2Saccharide identification of trestatin B, a small liquid ISP2 culture of *S. ansachromogenes* subsp. *pallens* NRRL B-12018 was cultivated and subsequently analyzed in its aqueous supernatant after ethyl acetate extraction for saccharides by LC-MS/MS. The *Streptomyces* extract was compared to an authentic standard of trestatins purified from a culture of the source organism *S. dimorphogenes* ATCC 31484. This authentic standard of trestatins included primarily trestatin A and B based on NMR analysis (Supporting Figures 7 and 8). In specific, the standard showed the δ­(^13^C) corresponding to the terminal α,α-1,1-glycosidic bond characteristic to trestatins (Supporting Figure 8 and Supporting Table 3). An analyte was detected in the extract of *S. ansachromogenes* subsp. *pallens* NRRL B-12018 that matched the Seq2Saccharide-identified trestatin B analyte in its precursor mass and tandem MS spectrum (Figure 7 and Supporting Figures 9 and 10). Furthermore, this analyte matched the authentic trestatin standard in retention time, MS and MS/MS data of its analyte corresponding to trestatin B. In addition, an analyte was characterized in the *S. ansachromogenes* sample which matched the trestatin A analyte in the authentic standard (Figure 7). This validated the identification of trestatin B in *S. ansachromogenes* subsp. *pallens* NRRL B-12018 by Seq2Saccharide and indicates that the predicted saccharide BGC in *S. ansachromogenes* subsp. *pallens* NRRL B-12018 encodes for the biosynthesis of trestatins A and B. Analysis of the putative trestatin gene cluster from *S. ansachromogenes* through MIBiG KnownClusterBlast revealed homology to the validamycin BGC reported from *Streptomyces hygroscopicus* ssp. *jinggangensis*. The similarity of both gene clusters is based on validamycin A sharing the valienamine monomer of trestatins (Supporting Figure 6). The identification of trestatins and their BGC from paired omics data sets showcases the applicability of Seq2Saccharide to discovery of saccharides and their biosynthetic pathways via scaled genome mining.

## Discussion

Pioneering genome mining approaches, like antiSMASH, have drastically changed the landscape of microbial natural product discovery. However, accurately predicting the structure of mature natural products from microbial genomics data remains a challenge. Computational methods that integrate genome mining with untargeted mass spectrometry are essential for the accurate prediction of the molecular structures of natural products.

Saccharides, despite their vast potential in biomedical applications, face significant challenges in the prediction of their chemical structures. This difficulty arises from the complexity of their biosynthesis including intricate modifications their backbones undergo. Existing tools fall short in predicting the correct monomer sets, fail to account for primary monomers during backbone construction, and struggle with handling postassembly modifications. To address these gaps, we introduce Seq2Saccharide, an innovative framework for discovering saccharide natural products from genomics and untargeted mass spectrometry data.

Seq2Saccharide offers several innovative enhancements over existing methodologies. It incorporates a sophisticated probabilistic model for predicting the set of monomers present in the molecule, significantly improving the accuracy of saccharide backbone prediction. Importantly, Seq2Saccharide considers primary monomers that are not present in the gene cluster. While this approach generates more candidate backbones, it ensures the correct backbone is identified by the algorithm. After applying postassembly modifications to the backbones, the candidate structures are filtered via mass spectral search, and the erroneous structures with low matching scores are filtered out. Seq2Saccharide also utilizes an in-house database of saccharide modifications constructed from literature surveys to accurately apply postassembly modifications to candidate backbones.

Seq2Saccharide significantly improves the accuracy of genome mining methods in predicting saccharide structures from BGCs. When analyzing 20 saccharide BGCs from the MIBiG database, Seq2Saccharide correctly identified 12 known saccharides without prior knowledge of the biosynthetic pathway, while PRISM failed to identify any. In PRISM’s results, only four molecules exhibited a Tanimoto similarity above 0.7. Further searches of these candidate structures against untargeted mass spectral data collected from microbial extracts, using Variable Dereplicator+, accurately identified five known saccharide BGCs from the GNPS *Streptomyces* data sets. Further, Seq2Saccharide identified a putative BGC for trestatin assembly.

The accuracy of Seq2Saccharide’s structure prediction is intrinsically dependent on the completeness of the input BGC, which is determined by its defined genomic boundaries. Given the dispersed nature of saccharide biosynthetic and tailoring genes, strict boundary definitions may omit essential enzymatic functions, whereas overly permissive boundaries risk incorporating unrelated genes and introducing noise. We therefore recommend that users critically evaluate and, if necessary, manually adjust BGC boundaries based on pathway context and gene content, particularly when predicted structures lack expected modifications or exhibit low structural diversity.

Seq2Saccharide demonstrates significantly higher prediction accuracy compared to existing tools like PRISM, but certain limitations prevent it from achieving perfect accuracy. These include challenges with saccharides containing large numbers of monomers (e.g., more than five), misidentification of modification genes, and unique reaction sites for postassembly modifications (Supporting Table 4). While introducing specific adjustments to address these cases could improve predictions for known molecules, it would reduce Seq2Saccharide’s ability to generalize and discover novel structures and would increase computational resource requirements. Seq2Saccharide can predict saccharides and aminoglycosides, which are saccharide-derived molecules composed of amino-modified sugar units. Currently Seq2Saccharide does not support hybrid molecules that contain polyketide and nonribosomal peptide moieties. As the number of available saccharides increases and our understanding of saccharide biosynthesis advances, we are confident that the algorithms for each step will improve and that any limitations will be addressed without compromising generalization or efficiency of Seq2Saccharide.

## Methods

Seq2Saccharide identified saccharide natural products through the following steps:

### Identification of Saccharide Biosynthetic Gene Clusters

Saccharide biosynthetic gene clusters (BGCs) consist of genes responsible for saccharide synthesis. To facilitate identification, Seq2Saccharide constructs Hidden Markov Models (HMMs) for each gene class involved in saccharide biosynthesis. Each input genome is aligned against these HMM motifs to identify all enzymes responsible for saccharide formation, using HMMER.^60^ Regions extending 10,000 base pairs (bp) upstream and downstream from these biosynthetic genes are designated as saccharide BGCs.

### Biosynthesis Pathway of Saccharide Monomers

Through a comprehensive literature review, we identified the genes involved in the biosynthesis of 20 unique monomers found in the known oligosaccharide BGCs listed in the MIBiG database (Supporting Figure 11). These genes were subsequently categorized based on their functional roles, and a Hidden Markov Model (HMM) was created for each gene category. To enhance identification accuracy, we incorporated similar genes from UniProt into the HMMs.

### Primary Saccharide Monomers

A literature survey identified six saccharide monomers involved in the primary metabolism of microbial species, which are sometimes unpredictable from genomic information. Seq2Saccharide accounts for the presence of d-mannose,^51^ d-glucose,^52^ d-glucosamine,^53^ *N*-acetylglucosamine,^54^ d-xylose,^51^ and d-ribose.^55^ This consideration occurs during the backbone construction process, even in the absence of corresponding biosynthesis genes within the BGC.

### Formation of Bonds between Monomers

After predicting the monomers in a saccharide from its BGC, Seq2Saccharide explores the possible bonds between different monomer pairs using a combinatorial approach. In the process of constructing saccharide structures, three fundamental rules are applied for bond formation between monomers, typically involving amidation, dehydration, glycosylation, and ribosylation reactions (Figure 2). Each bond formation is associated with the loss of a reactive group by each of the reacting monomers. The first and last monomers in the carbohydrate chain usually lose a single reaction group (as they form one bond), while all other monomers lose two reactive groups. To limit the number of possible backbones, Seq2saccharide currently does not support branching.

### Baseline Model for Selecting Set of Monomers

We assign a likelihood to the event that a given set of monomers being present in a saccharide produced by a BGC by examining the overlap between the genes detected in the BGC and the genes required for the synthesis of monomers in that set of genes. This strategy has previously been applied in the context of gene set enrichment analysis.^61^

Consider a total of *G* unique genes extracted for the production of saccharide monomers (*G* = 50 from our literature survey). Given a BGC annotated with *N* unique biosynthesis genes, a set of monomers requiring *M* genes for their biosynthesis, and an overlap of *O* genes between the two sets, Seq2Saccharide computes the *P*-value of Fisher’s exact test to determine the significance of the number of overlapping genes (Table 3).

$$P=\sum_{i\geq O}\frac{\left(\begin{array}{l} M\\ i \end{array}\right)\left(\begin{array}{l} G‐M\\ N‐i \end{array}\right)}{\left(\begin{array}{l} G\\ N \end{array}\right)}$$

**Table 3:** Contingency Table of Gene Set Enrichment Analysis for Genes Required by the Saccharide Biosynthesis and Genes Annotated in the BGC

#genes	annotated in BGC	not in BGC	row total
required by biosynthesis	O	M-O	M
not required	N–O	G-M-N + O	G-M
column total	N	G-N	G

### Probabilistic Model for Selecting a Set of Monomers

The baseline model cannot differentiate between missing and nonfunctional genes. Additionally, it cannot handle primary monomers for which biosynthesis genes are usually not found in the BGC. To overcome these limitations, Seq2Saccharide recruits a probabilistic model (Figure 8).

**Figure 8:**
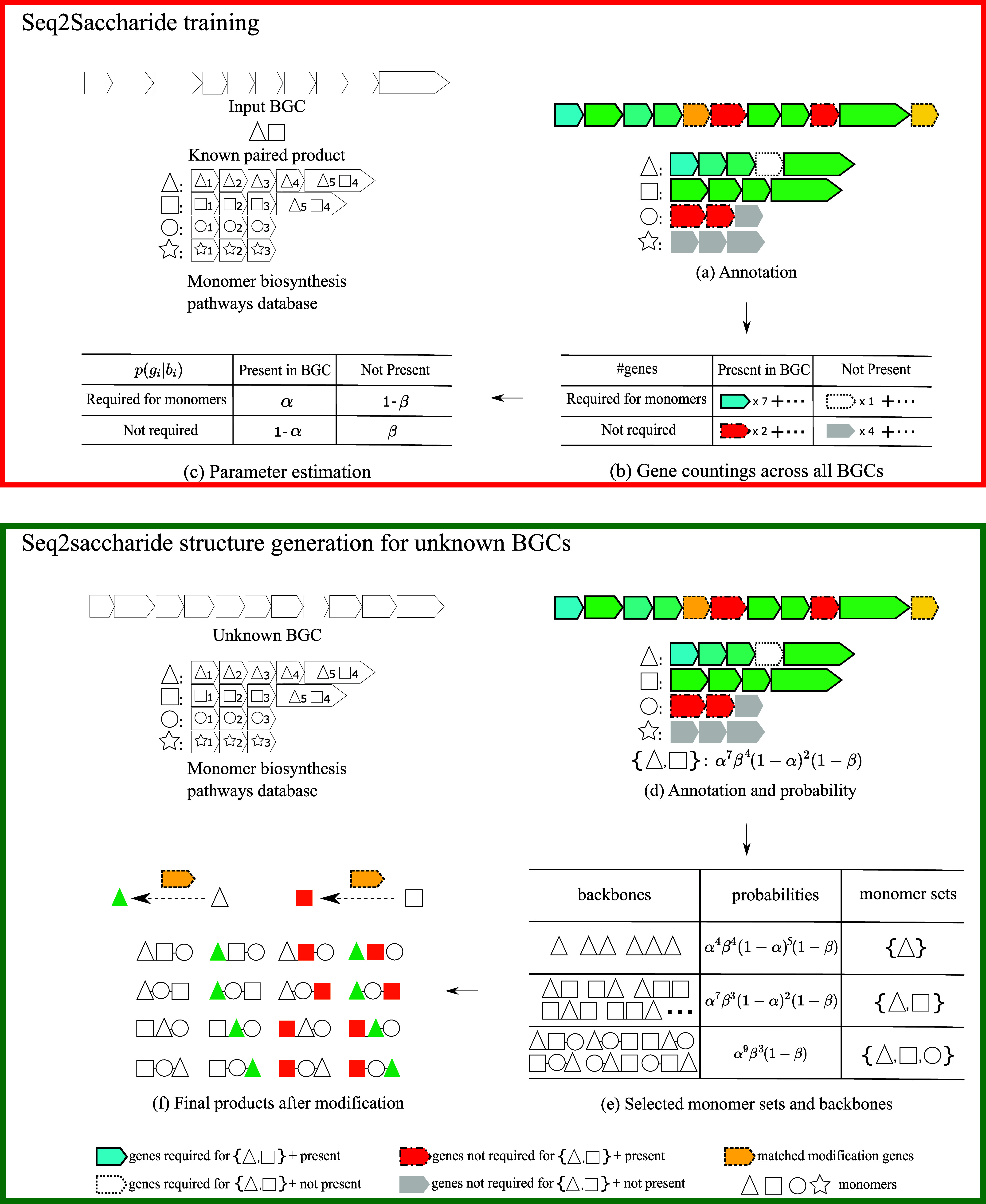
Pipeline for discovering saccharide natural products with a probabilistic model. During the training phase, for each pair of BGC and saccharide, (a) saccharide biosynthesis genes are labeled as follows: blue for required/present, blank for required/absent, red for unrequired/present, gray for unrequired/absent, and yellow for postassembly modification genes. Subsequently, (b) counts of each label type across all BGCs are aggregated, and (c) parameters for the monomer set prediction model are calculated. During the discovery process, for a detected BGC, (d) genes responsible for the biosynthesis of each monomer in the database are annotated. Monomers are considered produced by a BGC if at least one responsible gene is identified. Following this, (e) several monomer sets with the highest probabilities are selected based on the calculated probabilities, and all potential corresponding backbones are generated. (f) Final predicted products are then generated by applying possible modifications to each backbone.

Suppose there are *G* unique biosynthetic genes, and *K* unique primary monomers (*G* = 50 and *K* = 6). Seq2Saccharide assumes that biosynthetic genes are not required for primary monomers, while they are necessary for all other (secondary) monomers.

Seq2Saccharide represents BGCs as *G*-dimensional binary vector (*b*_1_, ···, *b*_*G*_), where *b*_*i*_ is a binary variable indicating whether the gene *i* is present in the BGC. Saccharides are represented by *G* + *K* dimensional binary vector (*g*_1_, ···,*g*_*G*_, *m*_1_, ···,*m*_*K*_), where *g*_*i*_ is a binary variable indicating whether gene *i* is required for biosynthesis of secondary monomers present in the saccharide, and *m*_*j*_ is a binary variable indicating whether the primary monomer *j* is required. Then assuming that whether a gene appears in the biosynthesis of a saccharide is independent of other genes given the BGC, we can obtain the probability of a BGC producing a saccharide as

$$\begin{array}{l} P\left(\mathrm{sacc}\mathrm{haride}|\mathrm{BGC}\right)=P\left(\left(g_{1},...,g_{G},m_{1},...,m_{K}\right)|\left(b_{1},...,b_{G}\right)\right)=\Pi_{i=1...G}P\left(g_{i}|b_{i}\right)\Pi_{j=1...K}P\left(m_{j}\right) \end{array}$$

where

$$P\left(g_{i}|b_{i}\right)=\{\begin{array}{ll} \alpha & \mathrm{if}\;g_{i}=1,b_{i}=1\\ 1-\alpha & \mathrm{if}\;g_{i}=0,b_{i}=1\\ 1-\beta & \mathrm{if}\;g_{i}=1,b_{i}=0\\ \beta & \mathrm{if}\;g_{i}=0,b_{i}=0 \end{array}$$

$$P\left(m_{j}\right)=\{\begin{array}{ll} \gamma & m_{j}=1\\ 1-\gamma & m_{j}=0 \end{array}$$

α, β, and γ are the parameters that need to be estimated from the training data. Let *N* denote the number of BGCs in the training data, and

$$\begin{array}{l} a=\#\left\{i|g_{i}=1,b_{i}=1\right\},b=\#\left\{i|g_{i}=0,b_{i}=1\right\},c=\#\left\{i|g_{i}=1,b_{i}=0\right\}\\ d=\#\left\{i|g_{i}=0,b_{i}=0\right\},e=\#\left\{i|m_{k}=1\right\},f=\#\left\{i|m_{k}=0\right\} \end{array}$$

Maximizing the log-likelihood function

L(α,β,γ)=log⁡Πn=1...NP(sacchariden|BGCn)=log⁡Πn=1...NP(sacchariden|BGCn)=log⁡Πn=1...NΠi=1...GP(gin|bin)Πj=1...KP(mkn)=log⁡αa(1−α)bβc(1−β)dγe(1−γ)f=a⁡log⁡α+b⁡log(1−α)+c⁡log⁡β+d⁡log(1−β)+e⁡log⁡γ+f⁡log(1−γ)

gives

$$\alpha =\frac{a}{a+b},\beta =\frac{c}{c+d},\gamma =\frac{e}{e+f}$$

### Creating the Postassembly Modification Database

Handling postassembly modifications in biosynthetic pathways presents a significant challenge due to their complexity and variability. These modifications often involve diverse enzymatic processes, such as glycosylation, methylation, and hydroxylation, which can occur in different orders and at multiple sites. The unpredictable nature of these modifications, coupled with the limited availability of annotated data sets, makes accurate prediction exceptionally difficult. Furthermore, the enzymatic machinery responsible for these modifications frequently exhibits substrate promiscuity, further complicating the modeling process. Existing computational tools often fail to incorporate the intricate network of dependencies between assembly line biosynthesis and subsequent modifications, leading to inaccuracies in predicting both the structure and function of the final product. This inherent complexity underscores the need for novel approaches capable of integrating domain-specific knowledge with robust computational frameworks to address these limitations effectively.

To address this challenge, Seq2saccharide utilizes a database of 49 saccharide postassembly modifications, compiled through literature mining. These postassembly modifications are stored in a computer-readable format (Figure 9), and the enzymes responsible for them are represented in HMM format. For each modification, the reaction motif is stored as a SMILES string, accompanied by a series of graph modifications (such as the addition or removal of nodes and/or edges) that are applied to the motif. These modifications are applied to the saccharide backbone structures whenever the corresponding enzymes are detected in the BGC.

**Figure 9:**
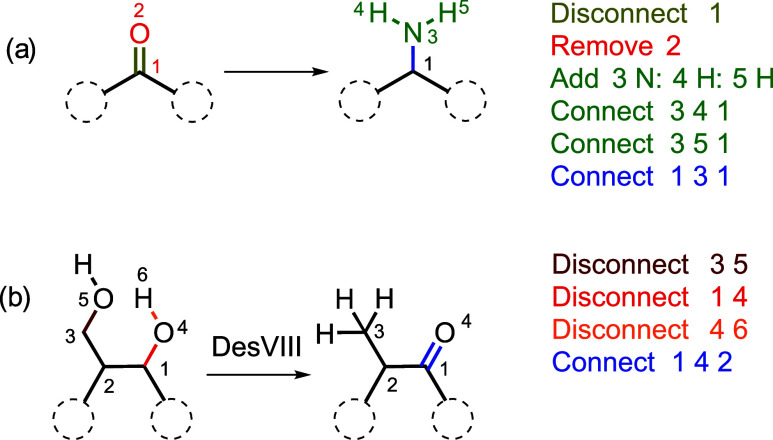
Enzymatic modifications expressed in a computer-readable format for (a) the aminotransferase enzyme (e.g., in sisomicin)^62^ and (b) the dehydratase enzyme (e.g., in desosamine).^63^ In this format, commands “add” and “remove” are used for atoms, while “disconnect” and “connect” are used for bonds. For example, in part (a), “disconnect 1 2” removes the bond between the carbon atom indexed 1 and the oxygen atom indexed 2. “remove 2” eliminates the oxygen atom indexed 2, while “add 3 N: 4 H: 5 H”, followed by “connect 3 4 1” and “connect 3 5 1” adds an NH_2_ amine group. Finally, “connect 1 3 1” forms a single bond between the carbon atom indexed 1 and the nitrogen atom indexed 3. In part (b), “connect 1 4 2” creates a double bond between the carbon atom indexed 1 and the oxygen atom indexed 4.

### *Streptomyces* Genome Assembly for Scaled Genome Mining

*S. ansachromogenes* ssp. *pallens* NRRL B-12018 was assembled from NCBI SRA data set SRR7783839 with BiosyntheticSPAdes and the assembly was annotated with Bakta. Saccharide BGCs in the assembled genome of *S. ansachromogenes* ssp. *pallens* NRRL B-12018 and the genome of *Streptomyces albofaciens* ATCC 23783 (GenBank GCA_008634025) were analyzed with antiSMASH, MIBiG and NCBI BLAST analysis.

### *Streptomyces* Cultivation and Metabolomic Analysis

All chemicals were obtained from Fisher Scientific unless otherwise noted. All solvents for extraction were HPLC grade, and all solvents for mass spectrometry analysis were LCMS (Thermo Optima) grade. *S. ansochromogenes* subspecies *pallens* NRRL B-12018 was obtained from the ARS Culture Collection of the United States Department of Agriculture. A 30 mL culture of *S. ansochromogenes* subspecies *pallens* NRRL B-12018 was inoculated from an ISP2 agar culture and cultivated in liquid ISP2 medium (4 g yeast extract, 10 g malt extract, 4 g d-glucose in 1 L deionized water, pH 7.3) for 11 days at 30 °C at 225 rpm.The medium was then centrifuged in a 50 mL centrifuge tube at 3200*g* for 30 min. The cell pellet and supernatant were separated, and the supernatant (30 mL) was extracted three times with 50 mL ethyl acetate to remove lipophilic components. The supernatant was dried by rotary evaporation at 40 °C. The dried supernatant was then resuspended in 3.5 mL deionized water, centrifuged for 5 min at 16,000*g*, and filtered with a Cytiva syringeless filter (0.2 μm mesh size, Cytiva, US503NPEORG). The filtered supernatant was immediately analyzed by liquid chromatography-tandem mass spectrometry (LC-MS/MS) on a Thermo QExactive Orbitrap mass spectrometer with a H-ESI-II ion source coupled to a Vanquish HPLC system.

The LC parameters were as follows: Phenomenex Kinetex 2.6 μm C18 reverse phase 100 Å 150 mm × 3 mm LC column, solvent A: 20 mM ammonium formate (pH 3.2), solvent B: 90% acetonitrile and 10% 200 mM ammonium formate (pH 3.2), column compartment temperature 30 °C, injection volume 5 μL, flow rate 0.5 mL/min, 0 min: 0% B, 5 min: 40% B, 4.5 min: 95% B, 5.5 min: 95% B, 6 min: 0% B, 10 min: 0% B. Electrospray ion source (Thermo H-ESI-II source) parameters were as follows: negative ion mode, sheath gas flow 35 psi, aux gas flow 3 psi, electrospray capillary temperature 320 °C, electrospray capillary voltage 3.6 kV. MS parameters were as follows: full MS, resolution 70,000; mass range 400–1200 *m*/*z*; dd-MS2 (data-dependent MS/MS), resolution 17,500; AGC target 1 × 10^5^, loop count 5, isolation width 1.0 *m*/*z*, collision energy 25 eV and dynamic exclusion 0.5 s. LC-MS data were analyzed with QualBrowser in the Thermo Xcalibur software package (v.4.3.73.11, Thermo Scientific).

### Isolation of Trestatins from a Culture of *S. dimorphogenes* ATCC 31484

*S. dimorphogenes* ATCC 31484 was grown on BTT agar plates [glucose (1%), yeast extract (0.1%), beef extract (0.1%), casein hydrolysate (0.2%), agar (1.5%), pH 7.4] at 30 °C for 3 days. A 1 cm^2^ section of mycelium was used to inoculate 100 mL of seed medium containing glucose (20 g/L), soytone (20 g/L), potato starch (20 g/L), yeast extract (2.5 g/L), NaCl (2.5 g/L), ZnSO_4_·7H_2_O (50 mg/L), CuSO_4_·5H_2_O (5 mg/L), MnCl_2_·4H_2_O (5 mg/L), and CaCO_3_ (3.2 g/L). The culture was shaken at 220 rpm at 28 °C for 3 days.

A 5 mL aliquot of the seed culture was then transferred to 100 mL of production medium with the same composition as the seed medium and cultured at 220 rpm, 28 °C, for 5 days. The culture broth was acidified to pH 4.0 using 1 M oxalic acid and centrifuged at 5000 rpm for 10 min. The supernatant was applied to a Dowex 50Wx2 (H^+^ form) column. The column was washed with 200 mL deionized water and eluted with 200 mL of 1.0 N NH_4_OH. The NH_4_OH fraction was lyophilized to yield a brown powder.

A portion of the powder (45 mg) was dissolved in 0.5 mL deionized water and subjected to Dowex 1x8 (OH^–^ form) column chromatography, eluted with deionized water. Fractions were monitored by TLC (silica gel F_254_, CHCl_3_–MeOH–25% NH_4_OH, 2:3:1) and visualized with CAM staining. Fractions containing trestatins (mostly trestatins A and B) were pooled and lyophilized to obtain a white powder (6.5 mg) (Supporting Figures 7, 8, 9, and 10).

## Supplementary Material



## Data Availability

The MS data sets used in this study are publicly available from the GNPS infrastructure under the following accession code: MSV00083738. LC-MS/MS data of aqueous fraction of *Streptomyces ansochromogenes* subspecies *pallens* NRRL B-12018 are deposited in GNPS under accession MSV000095359. Seq2Saccharide code are available from https://github.com/mohimanilab/Seq2Saccharide.

## Notes

Compound identifications, chemical structures, spectra, MS analysis, and biosynthetic pathways and candidate biosynthetic gene clusters, NMR comparisons, and prediction results from Seq2Saccharide (PDF)

## References

[1] Newman D. J., Cragg G. (2016). Natural products as sources of new drugs from 1981 to 2014. J. Nat. Prod..

[2] Tsunoda T., Samadi A., Burade S., Mahmud T. (2022). Complete biosynthetic
pathway to the antidiabetic drug acarbose. Nat.
Commun..

[3] Krause K. M., Serio A., Kane T., Connolly L. (2016). Aminoglycosides: an
overview. Cold Spring Harbor Perspect. Med..

[4] Flatt P. M., Mahmud T. (2007). Biosynthesis of aminocyclitol-aminoglycoside
antibiotics
and related compounds. Nat. Prod. Rep..

[5] Aanandhalakshmi, R. ; Sundar, K. ; Balakrishnan, V. Bioactive Oligosaccharides: Production, Characterization, and Applications; Nova Science Pub Inc., 2021; pp 165–199.

[6] Sato S., Fan P.-H., Yeh Y.-C., Liu H.-W. (2024). Complete In Vitro
Reconstitution of the Apramycin Biosynthetic Pathway Demonstrates
the Unusual Incorporation of a *β*-d-Sugar Nucleotide
in the Final Glycosylation Step. J. Am. Chem.
Soc..

[7] McDonald D., Hyde E., Debelius J., Morton J., González A., Ackermann G., Aksenov A., Behsaz B., Brennan C., Chen Y., Goldasich L. D., Dorrestein P., Dunn R., Fahimipour A., Gaffney J., Gilbert J., Gogul G., Green J., Hugenholtz P., Knight R. (2018). American Gut: an Open
Platform for Citizen Science
Microbiome Research. mSystems.

[8] Thompson L. R., Sanders J., McDonald D., Amir A., Ladau J., Locey K., Prill R., Tripathi A., Gibbons S., Ackermann G., Navas-Molina J., Janssen S., Kopylova E., Vázquez-Baeza Y., González A., Morton J., Mirarab S., Xu Z., Jiang L., Zhao H. (2017). A communal catalogue
reveals Earth’s multiscale microbial diversity. Nature.

[9] Integrative
HMP (iHMP) Research Network Consortium (2014). The Integrative Human Microbiome Project: Dynamic Analysis
of Microbiome-Host Omics Profiles during Periods of Human Health and
Disease. Cell Host Microbe.

[10] Wang M., Carver J. J., Phelan V. V., Sanchez L. M., Garg N., Peng Y., Nguyen D. D., Watrous J., Kapono C. A., Luzzatto-Knaan Tal. (2016). Sharing and community curation of mass
spectrometry data with Global Natural Products Social Molecular Networking. Nat. Biotechnol..

[11] Palaniappan K., Chen I-M. A., Chu K., Ratner A., Seshadri R., Kyrpides N. C., Ivanova N. N., Mouncey N. J. (2020). IMG-ABC v. 5.0:
an update to the IMG/Atlas of Biosynthetic Gene Clusters Knowledgebase. Nucleic Acids Res..

[12] Kautsar S. A., Blin K., Shaw S., Navarro-Muñoz J. C., Terlouw B. R., Van Der Hooft J. J. J., Van Santen J. A., Tracanna V., Duran H. G. S., Andreu V. P. (2020). MIBiG
2.0: a repository for biosynthetic gene clusters of known function. Nucleic Acids Res..

[13] Donia M., Cimermancic P., Schulze C., Brown L., Martin J., Mitreva M., Clardy J., Linington R., Fischbach M. (2014). A Systematic Analysis of Biosynthetic Gene Clusters
in the Human Microbiome Reveals a Common Family of Antibiotics. Cell.

[14] Skinnider M. A., Johnston C. W., Gunabalasingam M., Merwin N. J., Kieliszek A. M., MacLellan R. J., Li H., Ranieri M. R. M., Webster A. L. H., Cao My. (2020). Comprehensive prediction of secondary metabolite
structure and biological activity from microbial genome sequences. Nat. Commun..

[15] Blin K., Shaw S., Augustijn H., Reitz Z., Biermann F., Alanjary M., Fetter A., Terlouw B., Metcalf W., Helfrich E., Wezel G., Medema M., Weber T. (2023). antiSMASH
7.0: new and improved predictions for detection, regulation, chemical
structures and visualisation. Nucleic Acids
Res..

[16] Mohimani H., Gurevich A., Mikheenko A., Garg N., Nothias L.-F., Ninomiya A., Takada K., Dorrestein P. C., Pevzner P. A. (2017). Dereplication of peptidic natural
products through
database search of mass spectra. Nat. Chem.
Biol..

[17] Mohimani H., Gurevich A., Shlemov A., Mikheenko A., Korobeynikov A., Cao L., Shcherbin E., Nothias L.-F., Dorrestein P. C., Pevzner P. A. (2018). Dereplication of
microbial metabolites through database search of mass spectra. Nat. Commun..

[18] Cao L., Guler M., Tagirdzhanov A., Lee Y.-Y., Gurevich A., Mohimani H. (2021). MolDiscovery:
learning mass spectrometry fragmentation
of small molecules. Nat. Commun..

[19] Behsaz B., Mohimani H., Gurevich A., Prjibelski A., Fisher M., Vargas F., Smarr L., Dorrestein P. C., Mylne J. S., Pevzner P. A. (2020). De novo peptide
sequencing reveals
many cyclopeptides in the human gut and other environments. Cell Syst..

[20] Mohimani H., Kersten R. D., Liu W.-T., Wang M., Purvine S. O., Wu S., Brewer H. M., Pasa-Tolic L., Bandeira N., Moore B. S. (2014). Automated genome mining
of ribosomal peptide natural products. ACS Chem.
Biol..

[21] Cao L., Gurevich A., Alexander K. L., Naman C. B., Leão T., Glukhov E., Luzzatto-Knaan T., Vargas F., Quinn R., Bouslimani A. (2019). MetaMiner: a scalable peptidogenomics approach
for discovery of ribosomal peptide natural products with blind modifications
from microbial communities. Cell Syst..

[22] Lee Y.-Y., Guler M., Chigumba D., Wang S., Mittal N., Miller C., Krummenacher B., Liu H., Cao L., Kannan A., Narayan K., Slocum S., Roth B., Gurevich A., Behsaz B., Kersten R., Mohimani H. (2023). HypoRiPPAtlas
as an Atlas of hypothetical natural products for mass spectrometry
database search. Nat. Commun..

[23] Yan D., Zhou M., Adduri A., Zhuang Y., Guler M., Liu S., Shin H., Kovach T., Oh G., Liu X., Deng Y., Wang X., Cao L., Sherman D., Schultz P., Kersten R., Clement J., Tripathi A., Behsaz B., Mohimani H. (2024). Discovering type I cis-AT polyketides
through computational mass spectrometry and genome mining with Seq2PKS. Nat. Commun..

[24] Mohimani H., Liu W.-T., Kersten R. D., Moore B. S., Dorrestein P. C., Pevzner P. A. (2014). NRPquest: coupling
mass spectrometry and genome mining
for nonribosomal peptide discovery. J. Nat.
Prod..

[25] Behsaz B., Bode E., Gurevich A., Shi Y.-N., Grundmann F., Acharya D., Caraballo-Rodríguez A. M., Bouslimani A., Panitchpakdi M., Linck A. (2021). Integrating
genomics
and metabolomics for scalable non-ribosomal peptide discovery. Nat. Commun..

[26] Srivastava J., Sunthar P., Balaji P. V. (2021). Monosaccharide biosynthesis pathways
database. Glycobiology.

[27] Wehmeier, U. F. ; Piepersberg, W. Enzymology of Aminoglycoside BiosynthesisDeduction from Gene Clusters. In Methods in Enzymology; Elsevier, 2009; Vol. 459, pp 459–491. 19362651 10.1016/S0076-6879(09)04619-9

[28] McCranie E. K., Bachmann B. (2014). Bioactive Oligosaccharide Natural Products. Nat. Prod. Rep..

[29] Willett P., Barnard J., Downs G. (1998). Chemical similarity
searching. JChem
Inf Comput Sci. J. Chem. Inf. Comput. Sci..

[30] Rogers D. J., Tanimoto T. (1960). A computer program for classifying plants. Science.

[31] Kharel M. K., Pahari P., Shepherd M. D., Tibrewal N., Nybo S. E., Shaaban K. A., Rohr J. (2004). A gene cluster
for biosynthesis of
kanamycin from Streptomyces kanamyceticus: comparison with gentamicin
biosynthetic gene cluster. Arch. Biochem. Biophys..

[32] Veeneman G. H., van Boom J. H. (1991). Carbohydrate modifications:
Oxidation and methylation
of sugars. Tetrahedron Lett..

[33] Essentials of Glycobiology, 3rd ed.; Varki, A. ; Cummings, R. D. ; Esko, J. D. ; Stanley, P. ; Hart, G. W. ; Aebi, M. ; Darvill, A. G. ; Kinoshita, T. ; Packer, N. H. ; Prestegard, J. H. ; Schnaar, R. L. ; Seeberger, P. H. , Eds.; Cold Spring Harbor Laboratory Press, 2017. 27010055

[34] Glycobiology and Medicine; Ohtsubo, K. ; Marth, J. D. , Eds.; Springer, 2020.

[35] Park J. W., Park S. R., Nepal K., Han A., Ban Y., Yoo Y., Kim E., Kim E., Kim D., Sohng J. K., Yoon Y. (2011). Discovery of parallel pathways of
kanamycin biosynthesis allows antibiotic
manipulation. Nat. Chem. Biol..

[36] Weitnauer G., Mühlenweg A., Trefzer A., Hoffmeister D., Süssmuth R., Jung G., Welzel K., Vente A., Girreser U., Bechthold A. (2001). Biosynthesis of the orthosomycin
antibiotic avilamycin A: deductions from the molecular analysis of
the gene cluster. Chem. Biol..

[37] Wehmeier U. F., Piepersberg W. (2004). Biotechnology
and molecular biology of the *α*-glucosidase
inhibitor acarbose. Appl. Microbiol. Biotechnol..

[38] Piepersberg, W. ; Wehmeier, U. F. ; Kudo, F. ; Eguchi, T. The Biochemistry and Genetics of Aminoglycoside Producers. In Aminoglycoside Antibiotics; Wiley, 2007; pp 15–68.

[39] Kudo F., Numakura M., Tamegai H., Yamamoto H., Eguchi T., Kakinuma K. (2005). Extended sequence and
functional analysis of the butirosin
biosynthetic gene cluster. J. Antibiot..

[40] Dairi T., Ohta T., Hashimoto E., Hasegawa M. (1992). Organization and nature
of fortimicin A (astromicin) biosynthetic genes studied using a cosmid
library of Micromonospora olivasterospora DNA. Mol. Gen. Genet. MGG.

[41] Unwin J., Standage S., Alexander D., Hosted T. J., Horan A. C., Wellington E. M. H. (2004). Gene cluster in Micromonospora echinospora ATCC15835
for the biosynthesis of the gentamicin C complex. J. Antibiot..

[42] Palaniappan N., Ayers S., Gupta S., Habib E. S., Reynolds K. A. (2006). Production
of hygromycin A analogs in Streptomyces hygroscopicus NRRL 2388 through
identification and manipulation of the biosynthetic gene cluster. Chem. Biol..

[43] Clausnitzer D., Piepersberg W., Wehmeier U. F. (2011). The oxidoreductases LivQ and NeoQ
are responsible for the different 6’-modifications in the aminoglycosides
lividomycin and neomycin. J. Appl. Microbiol..

[44] Huang F., Haydock S. F., Mironenko T., Spiteller D., Li Y., Spencer J. B. (2005). The neomycin biosynthetic gene cluster of Streptomyces
fradiae NCIMB 8233: characterisation of an aminotransferase involved
in the formation of 2-deoxystreptamine. Org.
Biomol. Chem..

[45] Subba B., Kharel M., Lee H., Liou K., Kim B.-G., Sohng J. K. (2005). The Ribostamycin Biosynthetic Gene Cluster in Streptomyces
ribosidificus: Comparison with Butirosin Biosynthesis. Mol. Cells.

[46] Hong W. R., Ge M., Zeng Z. H., Zhu L., Luo M. Y., Shao L., Chen D. J. (2009). Molecular cloning
and sequence analysis of sisomicin
biosynthetic genes from Micromonospora inyoensis. Biotechnol. Lett..

[47] Kim K.-R., Kim T.-J., Suh J.-W. (2008). The Gene Cluster
for Spectinomycin
Biosynthesis and the Aminoglycoside-Resistance Function of spcM in
Streptomyces spectabilis. Curr. Microbiol..

[48] Kharel M. K., Basnet D. B., Lee H. C., Liou K., Woo J. S., Kim B.-G., Sohng J. K. (2004). Isolation
and characterization of
the tobramycin biosynthetic gene cluster from Streptomyces tenebrarius. FEMS Microbiol. Lett..

[49] Distler J., Mansouri K., Mayer G. (1996). Cloning and
expression analysis of
streptomycin biosynthesis genes from Streptomyces griseus. Arch. Microbiol..

[50] Takos, A. M. ; Lai, D. ; Mikkelsen, L. ; Abou Hachem, M. ; Shelton, D. ; Motawia, M. S. ; Olsen, C. E. ; Wang, T. L. ; Martin, C. ; Rook, F. ; Halkier, B. A. Genetic screening identifies cyanogenesis-deficient mutants of Lotus japonicus Sci. Rep. 2016; Vol. 6. 10.1105/tpc.109.073502 PMC2899875 20453117

[51] Thibodeaux C. J., Melançon C. E., Liu H.-w. (2008). Natural-Product
Sugar Biosynthesis and Enzymatic Glycodiversification. Angew. Chem., Int. Ed..

[52] Hodgson, D. A. Primary Metabolism and Its Control in Streptomycetes: A Most Unusual Group of Bacteria. In Advances in Microbial Physiology; Elsevier, 2000; Vol. 42, pp 47–238. 10907551 10.1016/s0065-2911(00)42003-5

[53] Moye Z. D., Burne R. A., Zeng L., Parales R. E. (2014). Uptake and Metabolism
of Acetylglucosamine and Glucosamine by Streptococcus mutans. Appl. Environ. Microbiol..

[54] Swiatek M., Tenconi E., Rigali S., van Wezel G. P. (2012). Functional
Analysis of the Acetylglucosamine Metabolic Genes of Streptomyces
coelicolor and Role in Control of Development and Antibiotic Production. J. Bacteriol..

[55] Mahoney D. E., Hiebert J., Thimmesch A., Pierce J., Vacek J., Clancy R., Sauer A., Pierce J. (2018). Understanding D-Ribose
and Mitochondrial Function. Adv. Biosci. Clin.
Med..

[56] Carroll, L. M. ; Larralde, M. ; Fleck, J. S. ; Ponnudurai, R. ; Milanese, A. ; Cappio, E. ; Zeller, G. Accurate de novo identification of biosynthetic gene clusters with GECCO bioRxiv 2021 10.1101/2021.05.03.442509. Submission Date: May 03, 2021.

[57] Kim S., Chen J., Cheng T., Gindulyte A., He J., He S., Li Q., Shoemaker B., Thiessen P., Yu B., Zaslavsky L., Zhang J., Bolton E. (2023). PubChem 2023 update. Nucleic Acids Res..

[58] Guo X., Geng P., Bai F., Bai G., Sun T., Li X., Shi L., Zhong Q. (2012). Draft genome sequence of streptomyces
coelicoflavus zg0656 reveals the putative biosynthetic gene cluster
of acarviostatin family a-amylase inhibitors. Lett. Appl. Microbiol..

[59] Tanoeyadi S., Tsunoda T., Ito T., Philmus B., Mahmud T. (2023). Acarbose may
function as a competitive exclusion agent for the producing bacteria. ACS Chem. Biol..

[60] Potter S. C., Luciani A., Eddy S., Park Y., Lopez R., Finn R. (2018). HMMER web server: 2018
update. Nucleic Acids
Res..

[61] Subramanian A., Tamayo P., Mootha V. K., Mukherjee S., Ebert B. L., Gillette M. A., Paulovich A., Pomeroy S. L., Golub T. R., Lander E. S., Mesirov J. P. (2005). Gene set
enrichment analysis: a knowledge-based approach for interpreting genome-wide
expression profiles. Proc. Natl. Acad. Sci.
U.S.A..

[62] Hong W.-R., Ge M., Zeng Z.-H., Zhu L., Luo M.-Y., Shao L., Chen D.-J. (2009). Molecular cloning and sequence analysis of the sisomicin
biosynthetic gene cluster from Micromonospora inyoensis. Biotechnol. Lett..

[63] Borisova S. A., Zhao L., Sherman D. H., Liu H.-w. (1999). Biosynthesis
of
desosamine: construction of a new macrolide carrying a genetically
designed sugar moiety. Org. Lett..

