# Supplementary material for: Seq2Saccharide: Discovering Oligosaccharides and Aminoglycosides Natural Products by Integrating Computational Mass Spectrometry and Genome Mining
Source: J Am Chem Soc. 2025 Sep 18;147(39):35323–38. doi: 10.1021/jacs.5c08251 (PMC12498427; doi:10.1021/jacs.5c08251)
Supplement: Supplementary file 1 [file ja5c08251_si_001.pdf]

Supplementary: Seq2Saccharide: Discovering oligosaccharides and aminoglycosides natural products by integrating computational mass spectrometry and genome mining

Donghui Yan †[1], Bahar Behsaz †[2], Yanjing Li †[1], Xiaofeng Wang †[3], Leigh Skala[4], Sitong Liu[1], Hyun Woo Lew[1], Mustafa Guler[1], Hunsica Jayaprakash[1], Muqing Zhou[1], Liu Cao[1], Ashootosh Tripathi[3,5,6], Jason A. Clement[7], Taifo Mahmud[4], Roland D. Kersten *[3], and Hosein Mohimani *[1]

[1]Computational Biology Department, School of Computer Science, Carnegie Mellon University, PA 15213, USA
[2]Chemia Biosciences, Pittsburgh, PA 15217, USA
[3]Department of Medicinal Chemistry, University of Michigan, Ann Arbor, MI 48109, USA
[4]Department of Pharmaceutical Sciences, Oregon State University, Corvallis, OR 97331, USA
[5]Natural Products Discovery Core, University of Michigan, Ann Arbor, MI 48109, USA
[6]Life Sciences Institute, University of Michigan, Ann Arbor, MI 48109, USA
[7]Baruch S. Blumberg Institute, 3805 Old Easton Road, Doylestown, PA 18902, USA

† These authors contributed equally to this work.
∗ Corresponding authors: rkersten@med.umich.edu, hoseinm@andrew.cmu.edu.

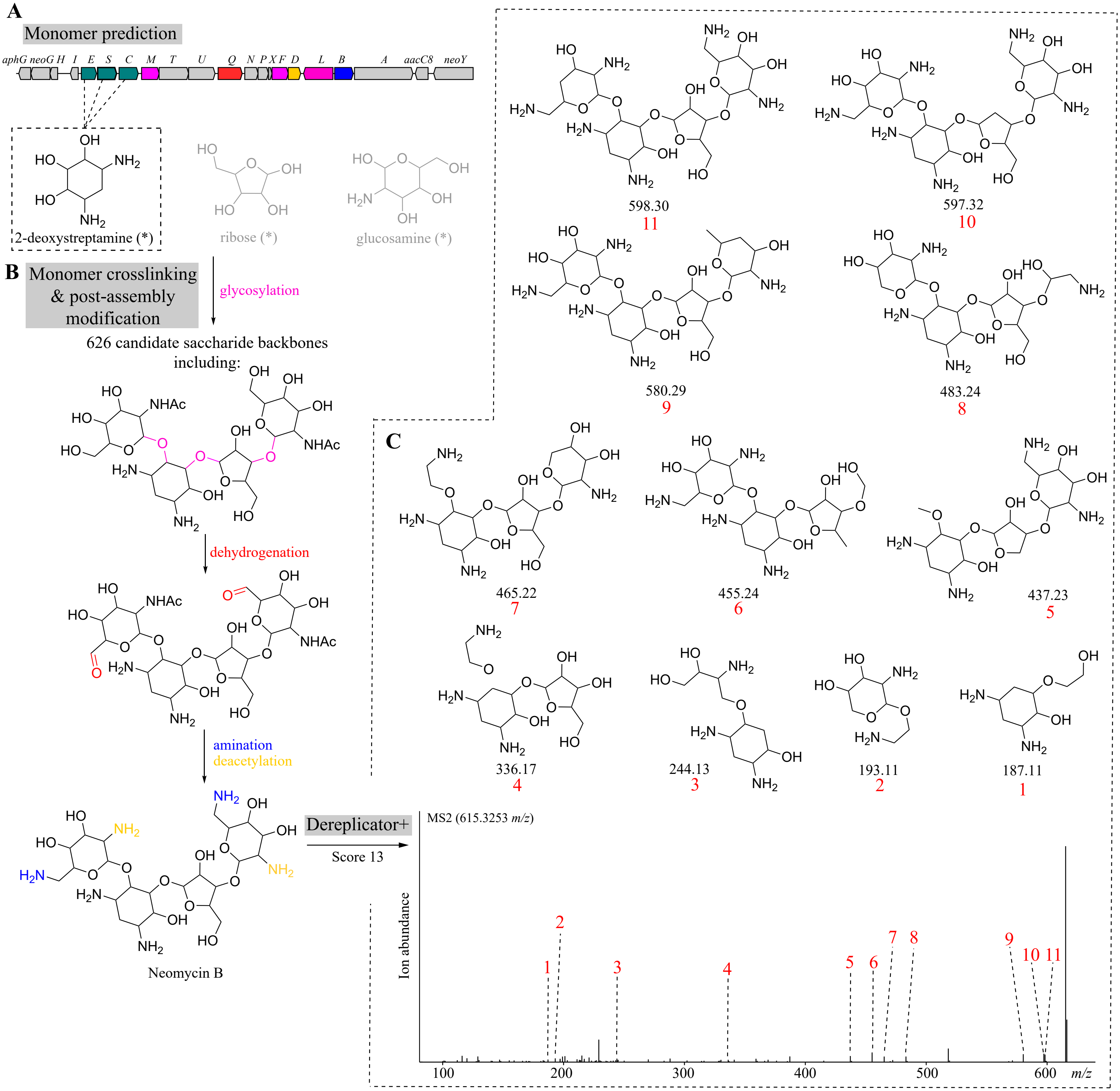

Supplementary Figure 1: Identification of neomycin B using Seq2Saccharide in *Streptomyces albogriseolus* ISP-5003. (A) Gene annotation results for neomycin BGC (MIBiG BGC0000711). Genes annotated in the genome are color-coded to indicate their roles predicted by Seq2Saccharide: green for 2-deoxystreptamine biosynthesis, pink for monomer crosslinking, red for monomer dehydrogenation, blue for monomer amination, orange for monomer deacetylation. Seq2Saccharide identified 3 potential genes involved in the formation of 2-deoxystreptamine. (**B**) Seq2Saccharide prediction of saccharide backbones from identified monomers including neomycin B. Seq2Saccharide predicts 2D structures (denoted by *) given the achiral nature of general tandem mass spectrometry data. (**C**) Dereplicator+ matching of neomycin B structure predicted from neomycin B gene cluster with tandem MS spectrum from *Streptomyces* extract LC-MS/MS dataset (GNPS-MassIVE MSV000088801, PLT2_D4.mzML, scan 961). Fragments from streptomycin and corresponding peaks in the tandem mass spectrum are annotated.

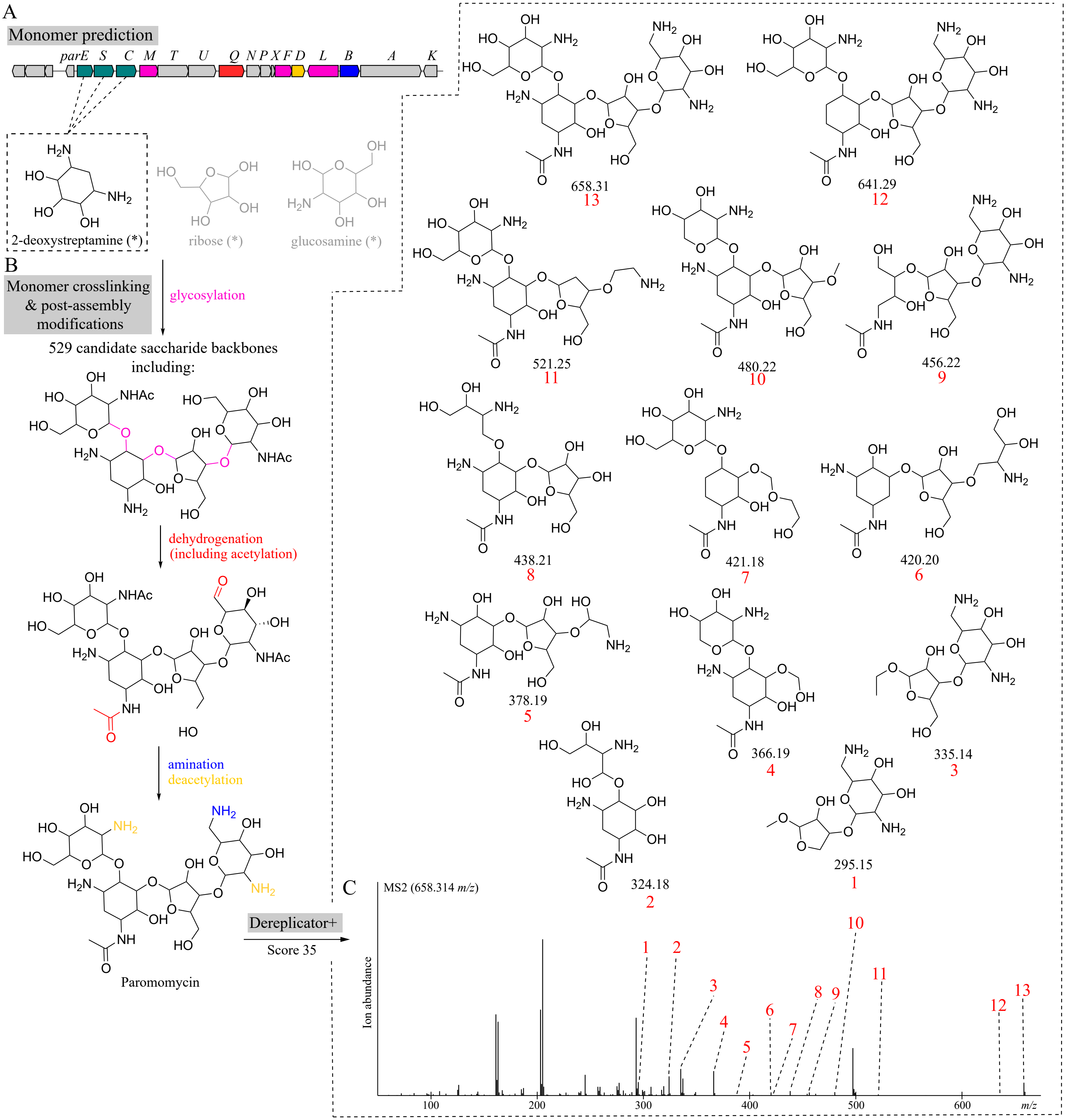

Supplementary Figure 2: Identification of paromomycin using Seq2Saccharide in *Streptomyces catenulae* ISP-5258. (**A**) Gene annotation results for paromomycin BGC (MIBiG BGC0000712). Genes annotated in the genome are color-coded to indicate their roles predicted by Seq2Saccharide: green for 2-deoxystreptamine biosynthesis, pink for monomer crosslinking, red for monomer dehydrogenation, blue for monomer amination, orange for monomer deacetylation. Seq2Saccharide identified 3 potential genes involved in the formation of 2-deoxystreptamine. (**B**) Seq2Saccharide prediction of saccharide backbone from identified monomers including paromomycin. Seq2Saccharide predicts 2D structures (denoted by *) given the achiral nature of general tandem mass spectrometry data. (**C**) Dereplicator+ matching of paromomycin structure predicted from paromomycin gene cluster with tandem MS spectrum from *Streptomyces* extract LC-MS/MS dataset (GNPS-MassIVE MSV000083738, WC3773_R8.mzML, scan 816). Fragments from paromomycin and corresponding peaks in the tandem mass spectrum are annotated.

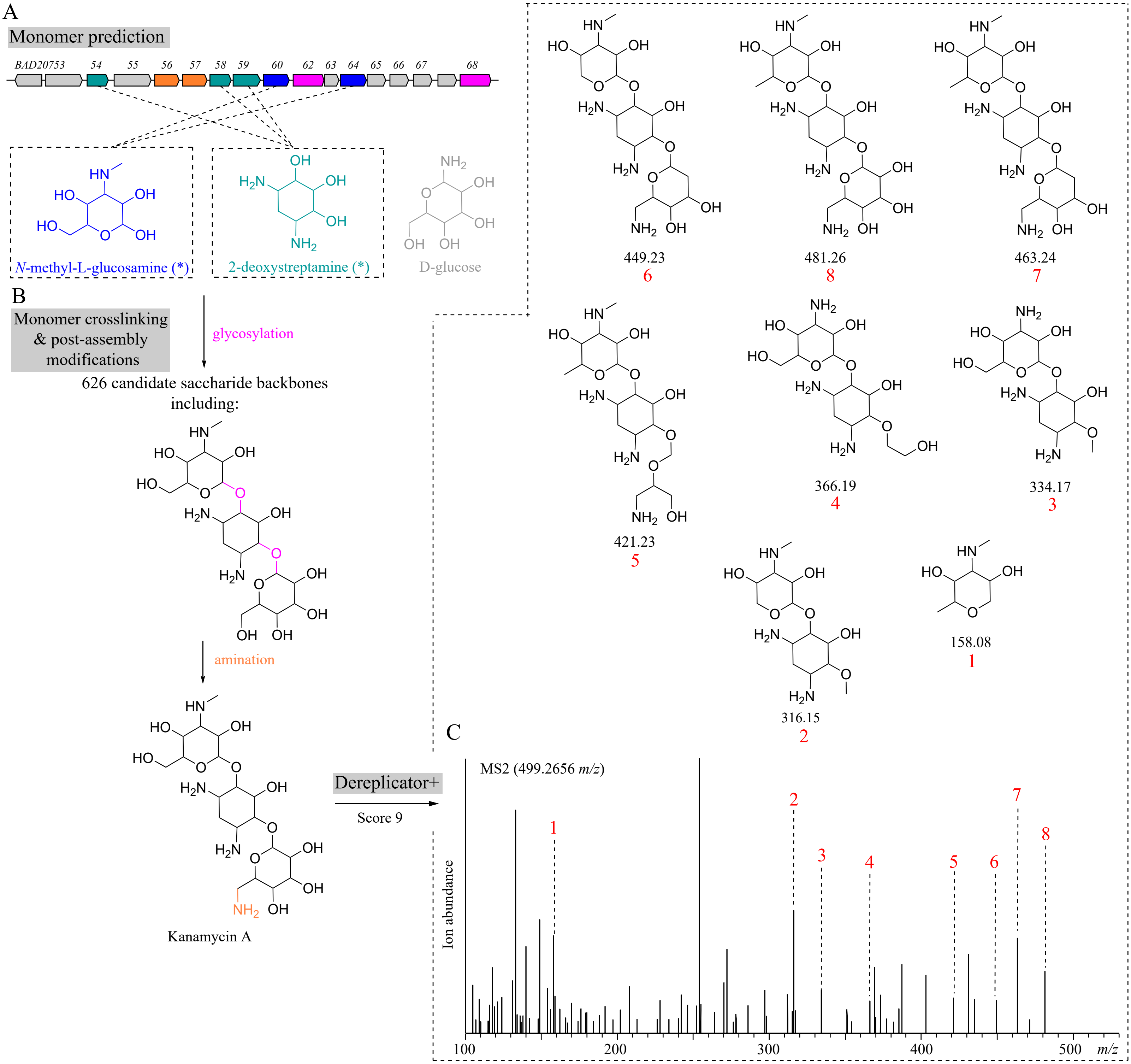

Supplementary Figure 3: Identification of kanamycin using Seq2Saccharide in *Streptomyces kanamyceticus* ISP-5500. (**A**) Gene annotation results for kanamycin BGC (MIBiG BGC0000702). Genes annotated in the genome are color-coded to indicate their roles predicted by Seq2Saccharide: green for 2-deoxystreptamine biosynthesis, blue for *N*-methyl-L-glucosamine biosynthesis, pink for monomer crosslinking, orange for monomer amination. Seq2Saccharide identified 3 potential genes involved in the formation of 2-deoxystreptamine and 2 potential genes for formation of N-methyl-L-glucosamine. (**B**) Seq2Saccharide prediction of saccharide backbones from identified monomers including kanamycin. Seq2Saccharide predicts 2D structures (denoted by *) given the achiral nature of general tandem mass spectrometry data. (**C**) Dereplicator+ matching of kanamycin structure predicted from kanamycin gene cluster with tandem MS spectrum from *Streptomyces* extract LC-MS/MS dataset (GNPS-MassIVE MSV000083738, WC3773_R8.mzML, scan 816). Fragments from kanamycin and corresponding peaks in the tandem mass spectrum are annotated.

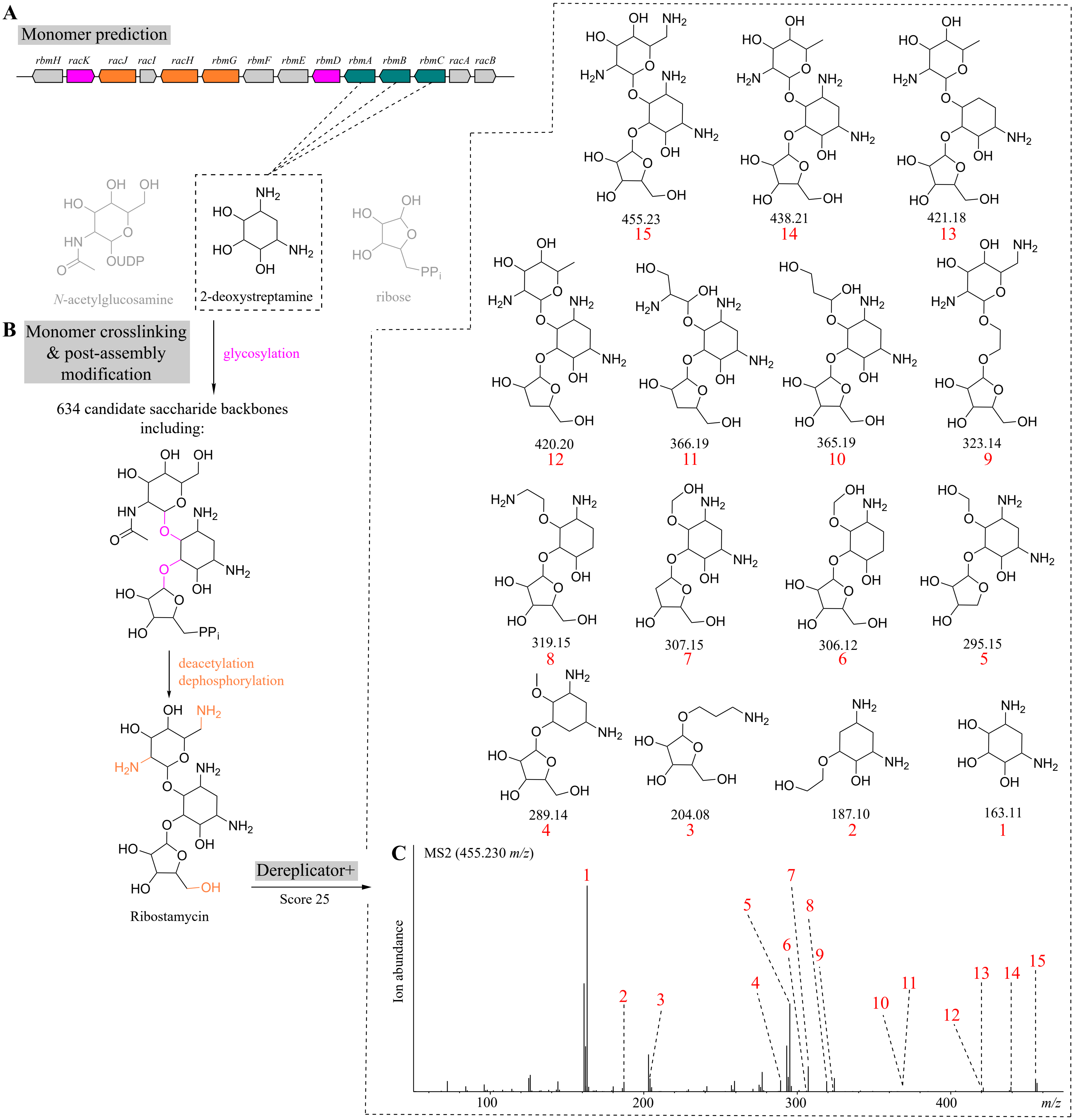

Supplementary Figure 4: Identification of ribostamycin using Seq2Saccharide in *Streptomyces ribosidificus* ATCC 21294. (**A**) Gene annotation results for ribostamycin BGC (MIBiG BGC0000713). Genes annotated in the genome are color-coded to indicate their roles predicted by Seq2Saccharide: green for 2-deoxystreptamine biosynthesis, pink for monomer crosslinking, orange for monomer deacetylation and monomer dephosphorylation. Seq2Saccharide identified 3 potential genes involved in the formation of 2-deoxy-streptamine. (**B**) Seq2Saccharide prediction of saccharide backbones from identified monomers including kanamycin. Seq2Saccharide predicts 2D structures (denoted by *) given the achiral nature of general tandem mass spectrometry data. (**C**) Dereplicator+ matching of ribostamycin structure predicted from ribostamycin gene cluster with tandem MS spectrum from *Streptomyces* extract LC-MS/MS dataset (GNPS-MassIVE MSV000083738, WC3773_R8.mzML, scan 812). Fragments from ribostamycin and corresponding peaks in the tandem mass spectrum are annotated.

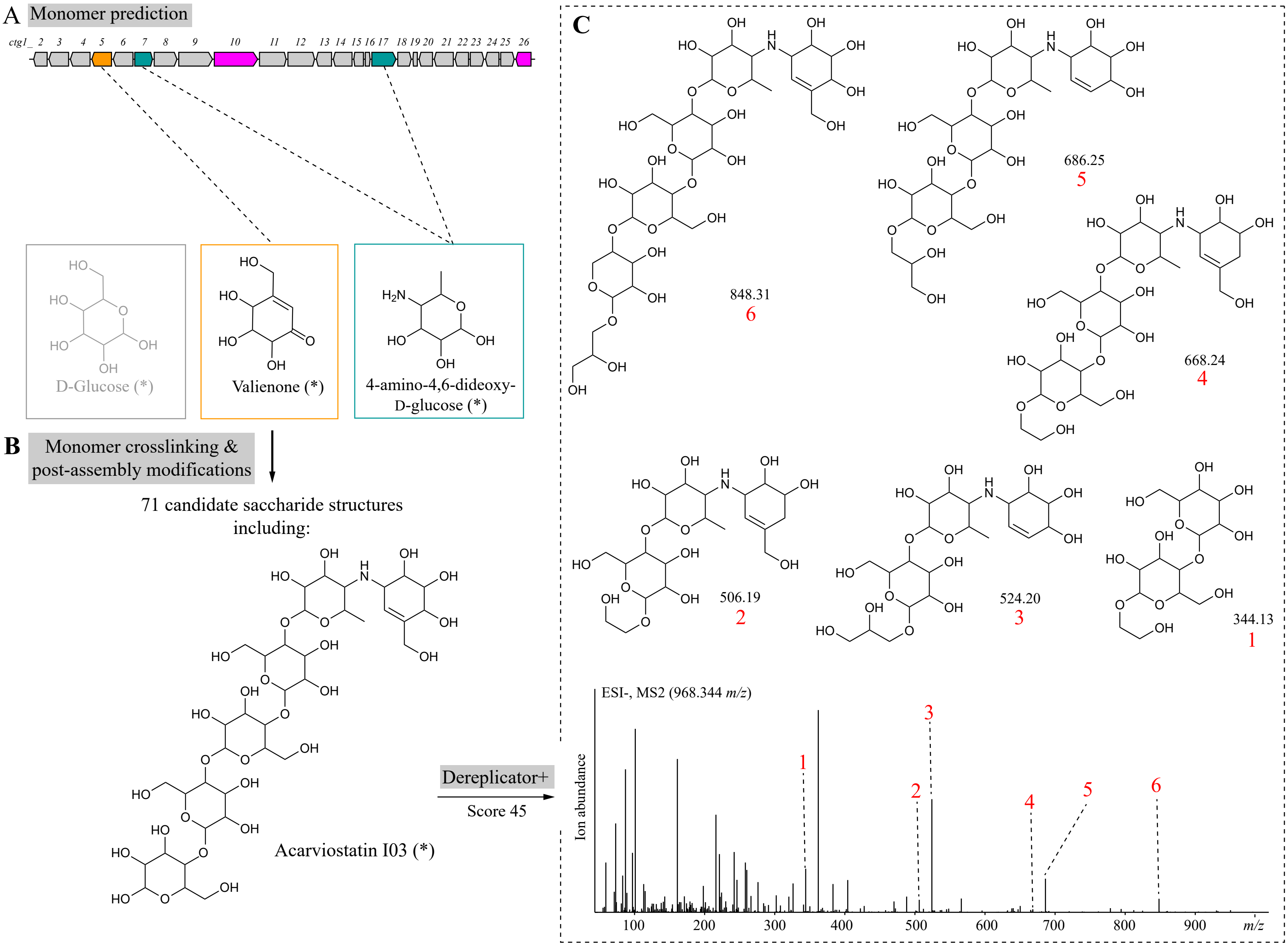

Supplementary Figure 5: Identification of acarviostatin I03 using Seq2Saccharide in *Streptomyces albofaciens* ATCC 23783. (**A**) Gene annotation results for putative acarviostatin BGC. Genes annotated in the genome are color-coded to indicate their roles predicted by Seq2Saccharide: green for 4-amino-4,6-dideoxy-D-glucose biosynthesis, orange for valienone biosynthesis, pink for monomer crosslinking. Seq2Saccharide identified one potential gene involved in the formation of valienone and two potential genes involved in 4-amino-4,6-dideoxy-D-glucose. (**B**) Seq2Saccharide prediction of saccharide backbones from identified monomers including kanamycin. Seq2Saccharide predicts 2D structures (denoted by *) given the achiral nature of general tandem mass spectrometry data. (**C**) Dereplicator+ matching of acarviostatin I03 structure predicted from acarviostatin I03 gene cluster with tandem MS spectrum from *Streptomyces* extract LC-MS/MS dataset (GNPS-MassIVE MSV000097846, 12172.mzXML, scan 20738). Fragments from acarviostatin I03 and corresponding peaks in the tandem mass spectrum are annotated.

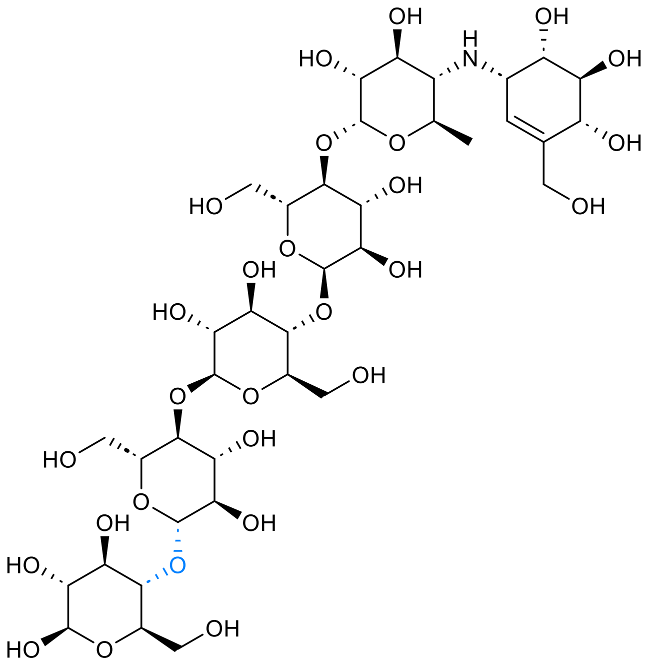

Acarviostatin I03

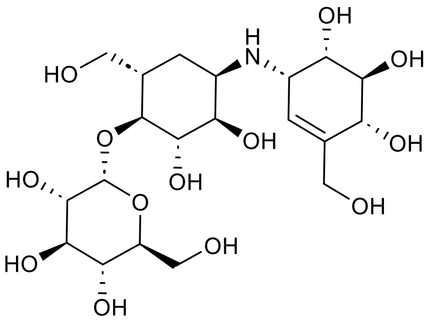

Validamycin A

Supplementary Figure 6: Chemical structures of acarviostatin I03 and validamycin A. The bond at the terminal disaccharide unit in acarviostatin I03 is labeled as the difference to trestatin B.

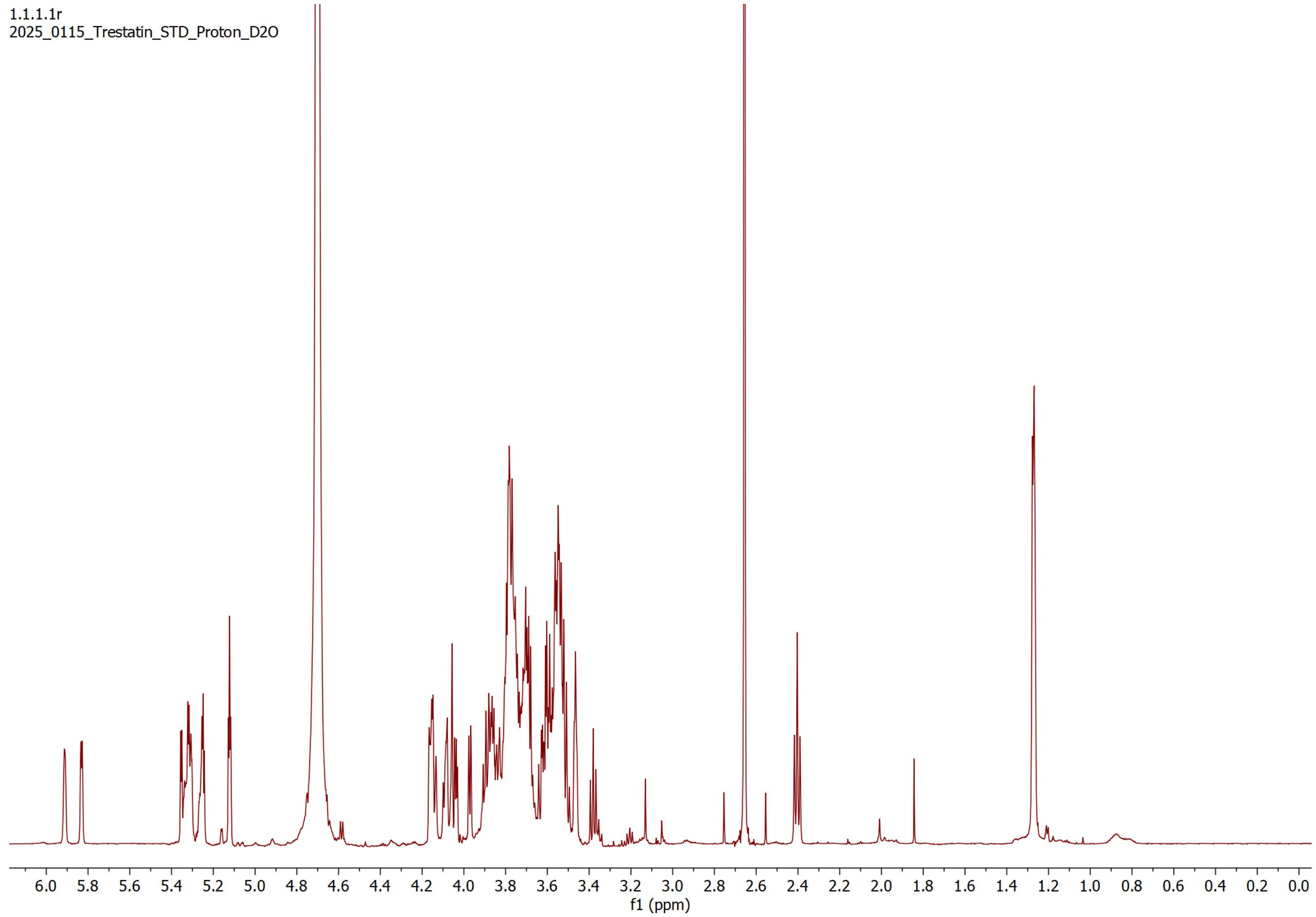

Supplementary Figure 7: ^{1}H-NMR spectrum of trestatin standard in D_2O (700 MHz).

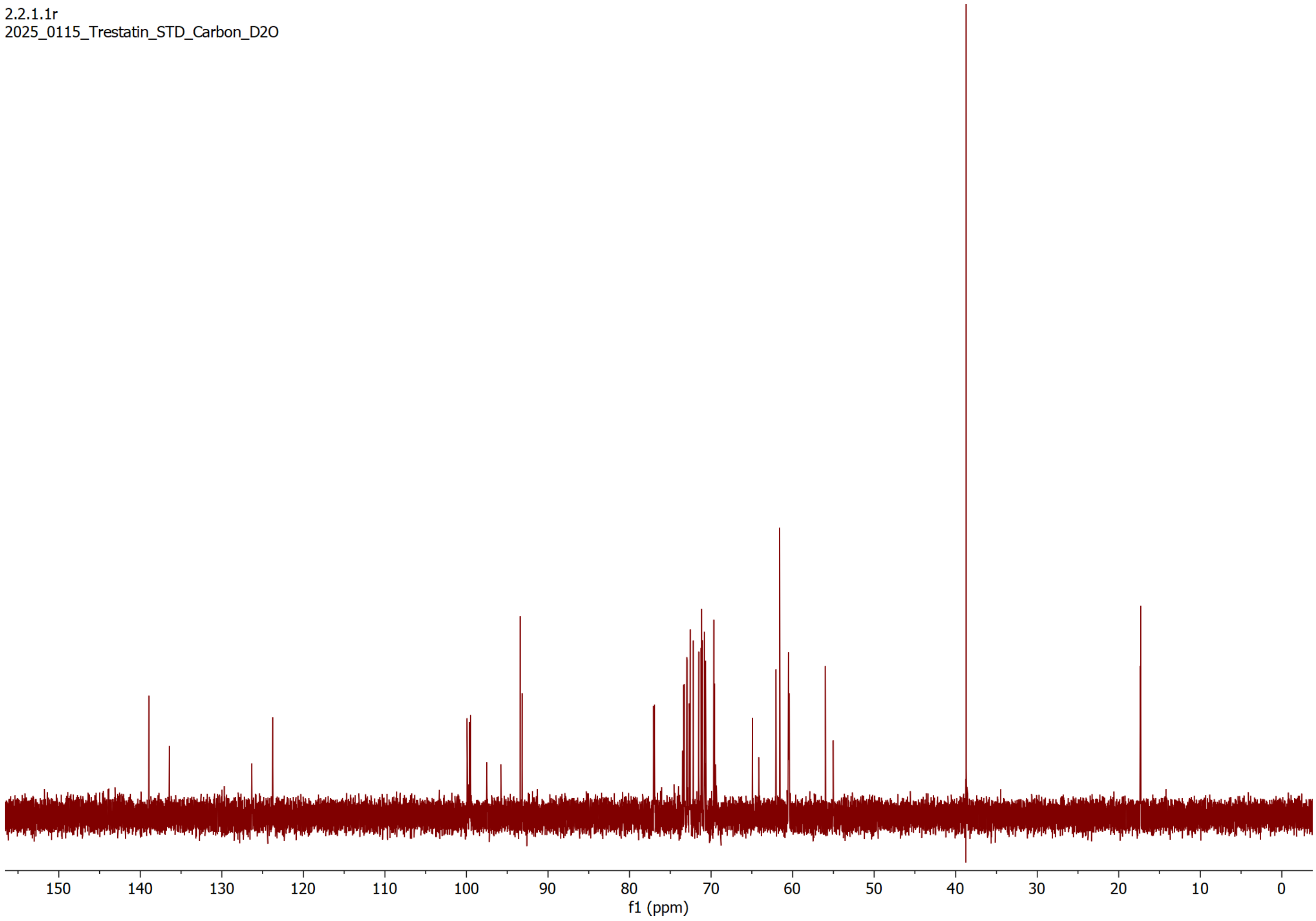

Supplementary Figure 8: ^{13}C-NMR spectrum of trestatin standard in D_2O (176 MHz).

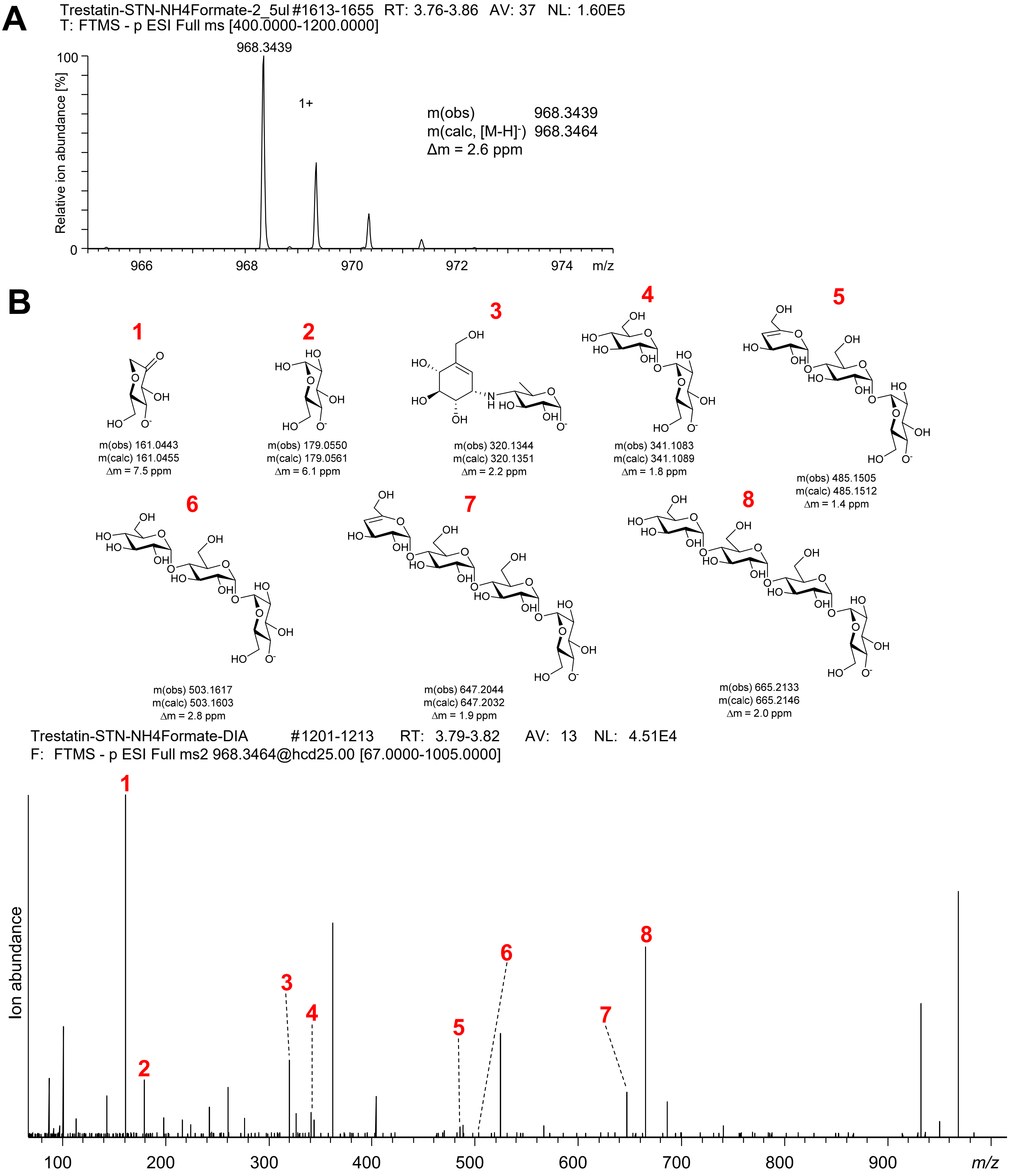

Supplementary Figure 9: Mass spectrometry (MS) analysis of trestatin standard from *Streptomyces dimorphogenes* ATCC 31484. (**A**) MS signal (negative ion mode) of trestatin B in trestatin standard isolated from *Streptomyces dimorphogenes* ATCC 31484. (**B**) Tandem mass spectrum of trestatin B analyte in trestatin standard isolated from *Streptomyces dimorphogenes* ATCC 31484 and annotated fragment masses with putative trestatin B substructures.

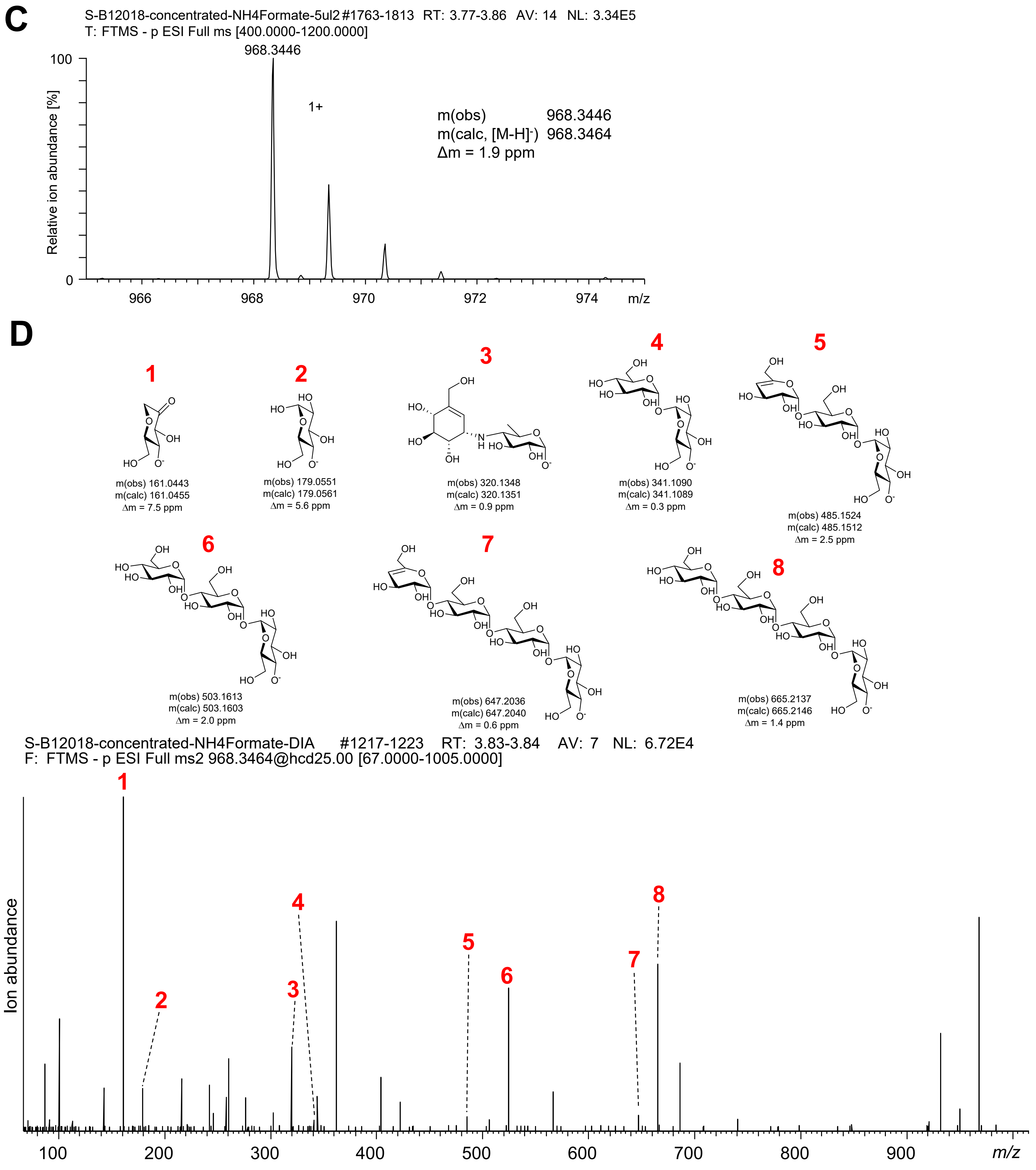

Supplementary Figure 10: Mass spectrometry (MS) analysis of putative trestatin B analyte from *Streptomyces ansochromogenes* subsp. *pallens* B-12018. (**A**) MS signal (negative ion mode) of trestatin B analyte in aqueous *S. ansochromogenes* subsp. *pallens* B-12018 medium. (**D**) Tandem mass spectrum of trestatin B analyte in in aqueous *S. ansochromogenes* subsp. *pallens* B-12018 medium and annotated fragment masses with putative trestatin B substructures.

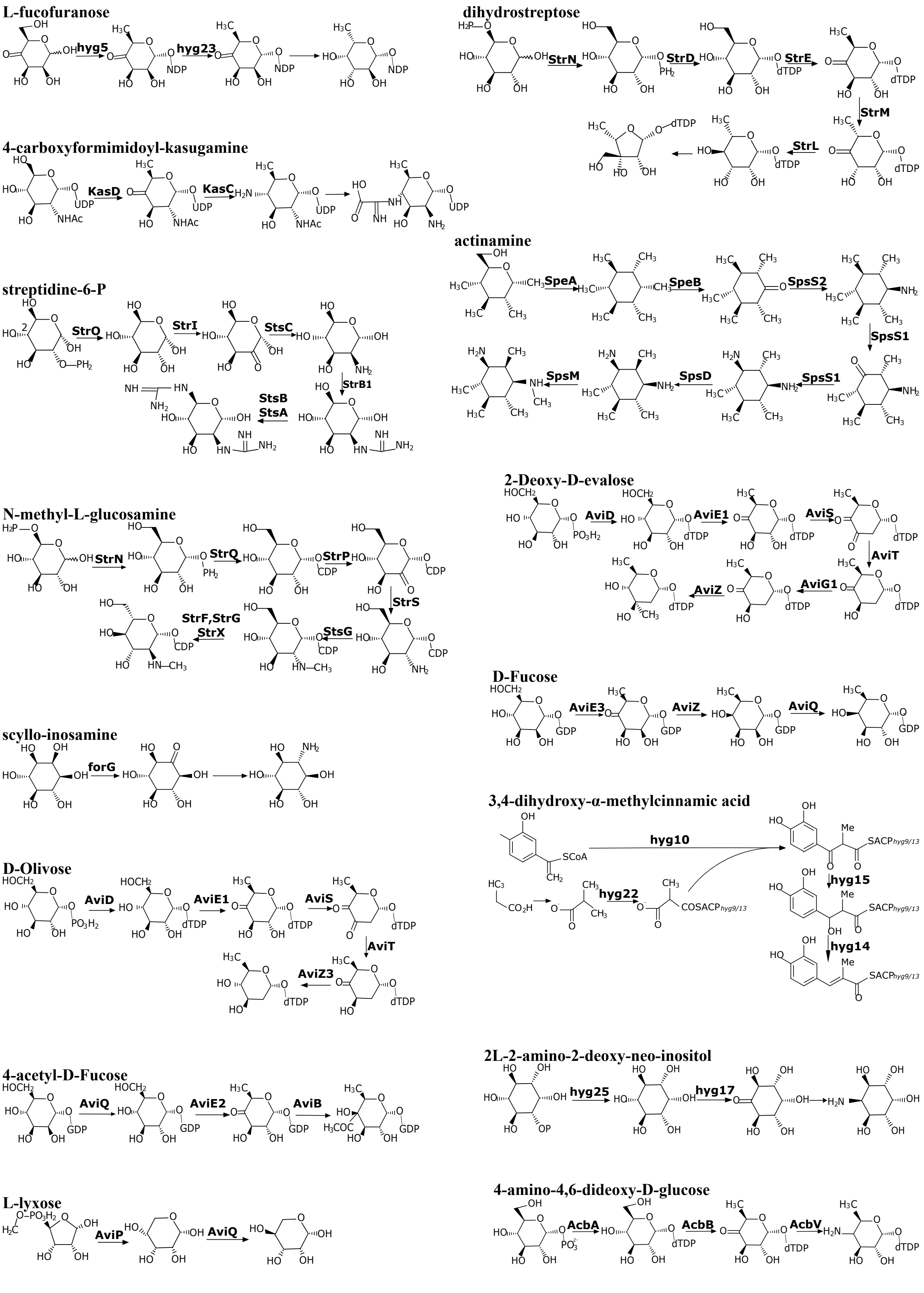

Supplementary Figure 11. The biosynthesis pathway for saccharide monomers along with the required genes.

Supplementary Table 1 | Candidate biosynthetic gene cluster of trestatins identified by Seq2Saccharide in *Streptomyces ansachromogenes* subsp. *pallens* NRRL B-12018. Gene highlighted in green was identified by Seq2Saccharide for 4-amino-4,6-dideoxy-D-glucose prediction, gene highlighted in orange was identified by Seq2Saccharide for valienone prediction.

Name	Description	length [nt]	length [aa]	MIBiG Homolog	MIBiG cluster product	MIBiG cluster
ctg21_13	ParB/RepB/Spo0J family partition protein	1050	349	BAG22760.1 | streptomycin biosynthesis operon regulator (50% ID)	streptomycin	BGC0000724
ctg21_14	hypothetical protein	684	227	n/a	n/a	n/a
ctg21_15	alcohol dehydrogenase catalytic domain-containing protein	891	296	n/a	n/a	n/a
ctg21_16	hypothetical protein	330	109	n/a	n/a	n/a
ctg21_17	hypothetical protein	549	182	n/a	n/a	n/a
ctg21_18	hypothetical protein	936	311	n/a	n/a	n/a
ctg21_19	sugar ABC transporter substrate-binding protein	255	84	n/a	n/a	n/a
ctg21_20	hypothetical protein	372	123	n/a	n/a	n/a
ctg21_21	hypothetical protein	420	139	n/a	n/a	n/a
ctg21_22	glycosyltransferase	1044	347	AXH03913.1 | polysaccharide biosynthesis protein (38%)	Heteropoly-saccharide (HePS)	BGC0001894
ctg21_23	sedoheptulose 7-phosphate cyclase	1224	407	ABL74381.1 | 2-epi-5-epi-valiolone synthase (64%)	cetoniacytone A	BGC0000283
ctg21_24	cupin domain-containing protein	747	248	ACH85570.1 | CetJ1 (56% ID)	cetoniacytone A	BGC0000283
ctg21_25	Mannose-6-phosphate isomerase	933	310	ABL74382.1 | putative glyoxalase/bleomycin resistance protein (62% ID)	cetoniacytone A	BGC0000283
ctg21_26	HAD-IA family hydrolase	744	247	ABC67272.1 | VldH (38% ID)	validamycin A	BGC0000723
ctg21_27	SDR family NAD(P)-dependent oxidoreductase	768	255	CAA09651.1 | 2,3-dihydro-2,3-dihydroxybenzoate dehydrogenase; RBL00455 (34% ID)	Fuscachelin A	BGC0000359
ctg21_28	glycosyltransferase	1338	445	ABC67275.1 | putative glycosyltransferase (54% ID)	validamycin A	BGC0000723
ctg21_29	MFS transporter	1194	397	ABC67274.1 | putative transport protein (60% ID)	validamycin A	BGC0000723
ctg21_30	MFS transporter	1242	413	QTK22484.1 | major facilitator superfamily (MFS) transporter (38% ID)	coformycin	BGC0002462
ctg21_31	NUDIX domain-containing protein	2490	829	EHN79459.1 | glycosyltransferase ScatS (40% ID)	acarviostatin	BGC0000804
ctg21_32	hypothetical protein	411	137	WP_381562631.1 | hypothetical protein [Streptomyces eurythermus] (100/100)	n/a	n/a
ctg21_33	sugar phosphate nucleotidyltransferase	1167	389	WP_381562630.1 | glucose-1-phosphate adenylyltransferase family protein [Streptomyces eurythermus] (100/100)	n/a	n/a
ctg21_34	ROK family protein	1095	365	WP_388036109.1 | ROK family protein [Streptomyces eurythermus] (100/100)	n/a	n/a
ctg21_35	SDR family oxidoreductase	909	303	WP_388036106.1 | SDR family oxidoreductase [Streptomyces eurythermus] (99/99)	n/a	n/a
ctg21_36	glycosyltransferase	1230	410	WP_381562627.1 | glycosyltransferase [Streptomyces eurythermus] (100/99)	n/a	n/a
ctg21_37	isopenicillin N synthase family dioxygenase	972	324	WP_381562626.1 | isopenicillin N synthase family dioxygenase [Streptomyces eurythermus] (100/99)	n/a	n/a
ctg21_38	L-glyceraldehyde 3-phosphate reductase	936	312	WP_381562625.1 | aldo/keto reductase [Streptomyces eurythermus] (100/99)	n/a	n/a
ctg21_39	MarR family transcriptional regulator	516	172	WP_388036096.1 | MarR family transcriptional regulator (100/100)	n/a	n/a
ctg21_40	Blue pigment (Indigoidine) exporter	1002	334	WP_388036099.1 | DMT family transporter [Streptomyces eurythermus] (100/99)	n/a	n/a
ctg21_41	n/a	108	36	n/a	n/a	n/a
ctg21_42	endoglucanase	2538	846	QIS69649.1 | endoglucanase [Streptomyces sp. DSM 40868] (97/96)	n/a	n/a
ctg21_43	hypothetical protein	156	52	WP_381562621.1 | hypothetical protein [Streptomyces eurythermus] (98/98)	n/a	n/a
ctg21_44	LysR family transcriptional regulator	954	318	WP_388036795.1 | LysR family transcriptional regulator [Streptomyces eurythermus] (100/99)	n/a	n/a
ctg21_45	hypothetical protein	216	72	WP_381742242.1 | hypothetical protein [Streptomyces andamanensis] (69/56)	n/a	n/a

Supplementary Table 2 | Candidate biosynthetic gene cluster of trestatins identified by Seq2Saccharide in *Streptomyces albofaciens* ATCC 23783. Gene highlighted in green was identified by Seq2Saccharide for 4-amino-4,6-dideoxy-D-glucose prediction, gene highlighted in orange was identified by Seq2Saccharide for valienone prediction.

Name	Description	length [nt]	length [aa]	MIBiG Homolog	MIBiG cluster product	MIBiG cluster
ctg1_1	choline_dehydrogenase	324	108	AEF33092.1 | choline_dehydrogenase (38% ID)	pyridomycin	BGC0001039
ctg1_2	NAD-dependent epimerase/dehydratase	939	312	EHN79433.1 | putative_2-epi-5-epi-valiolone_dehydratase/epimerase_ScatO (65% ID)	acarviostatin I03	BGC0000804
ctg1_3	ROK family protein	1041	346	EHN79466.1 | C7-cyclitol-7-kinase_ScatM (73% ID)	acarviostatin I03	BGC0000804
ctg1_4	none	384	127	EHN79465.1 | ScatJ_protein (78% ID)	acarviostatin I03	BGC0000804
ctg1_5	3-dehydroquinate synthase	1245	414	EHN79464.1 | 2-epi-5-epi-valiolone_synthase_ScatC (74% ID)	acarviostatin I03	BGC0000804
ctg1_6	none	2076	691	EHN79463.1 | putative_acarbose_4-alpha-glucanotransferase_ScatQ (73% ID)	acarviostatin I03	BGC0000804
ctg1_7	hydrolase	3060	1019	EHN79462.1 | putative_glycosyltransferase_ScatI (64% ID)	acarviostatin I03	BGC0000804
ctg1_8	PfkB domain protein	951	316	EHN79461.1 | acarbose-7-kinase_ScatK (75% ID)	acarviostatin I03	BGC0000804
ctg1_9	glucose-1-phosphate adenylyl/thymidylyltransferase	1107	368	EHN79460.1 | putative_1-epi-valienol-1,7-bisphosphate-1-adenylyltransferase_ScatR (72% ID)	acarviostatin I03	BGC0000804
ctg1_10	glycosyl transferase group 1	2070	689	EHN79459.1 | glycosyltransferase_ScatS (76% ID)	acarviostatin I03	BGC0000804
ctg1_11	none	1452	483	EHN79458.1 | putative_kinase_(1-epi-valienol-7-phosphate-1-kinase)_ScatU (59% ID)	acarviostatin I03	BGC0000804
ctg1_12	none	801	266	EHN79457.1 | ABC_transporter_permease_protein_ScatY (82% ID)	acarviostatin I03	BGC0000804
ctg1_13	none	861	286	EHN79456.1 | ABC_transporter_permease_protein_ScatX (80% ID)	acarviostatin I03	BGC0000804
ctg1_14	ABC transporter ATP-binding protein	1047	348	EHN79455.1 | ABC_transporter_ATP-binding_protein_ScatW (85% ID)	acarviostatin I03	BGC0000804
ctg1_15	aminotransferase class-III	1293	430	EHN79454.1 | dTDP-4-keto-6-deoxy-glucose_4-aminotransferase_ScatV (78% ID)	acarviostatin I03	BGC0000804
ctg1_16	glucose-1-phosphate adenylyl/thymidylyltransferase	1077	358	EHN79432.1 | dTDP-glucose_synthase_ScatA (72% ID)	acarviostatin I03	BGC0000804
ctg1_17	NAD-dependent epimerase/dehydratase	978	325	EHN79431.1 | dTDP-glucose_4,6-dehydratase_ScatB (75% ID)	acarviostatin I03	BGC0000804
ctg1_18	Alpha-glucosidase	1881	626	EHN79425.1 | alpha-amylase_ScatE2 (75% ID)	acarviostatin I03	BGC0000804
ctg1_19	Pullulanase, type I	3147	1048	EHN79424.1 | alpha-amylase_ScatZ1 (67% ID)	acarviostatin I03	BGC0000804
ctg1_20	none	1809	602	AGU42410.1 | arabinofuranosidase (46% ID)	carbapenem MM4550	BGC0000842
ctg1_21	none	720	239	ARO49588.1 | 1,4-beta-xylanase (58% ID)	belactosin A	BGC0001441
ctg1_22	sensor histidine kinase	1125	374	AFU82609.1 | 2_component_system_sensor_kinase (44% ID)	guadinomine	BGC0000998
ctg1_23	LuxR family DNA-binding response regulator	672	223	AFU82608.1 | 2_component_system_sensor_kinase (59% ID)	guadinomine	BGC0000998
ctg1_24	none	330	109	n/a		
ctg1_25	none	240	79	n/a		
ctg1_26	glycosyl transferase group 1	2127	708	ADD45304.1 | glycosyl_transferase_group_1	phosphonoglycans	BGC0000807

Supplementary Table S3: Comparison of NMR spectral data in D_2O for the trestatin standard and literature-reported trestatin A and B [1].

		Trestatin standard	Reference Trestatin A		Trestatin standard	Reference Trestatin B	
Position		δ(^{13}C) [ppm] (176 MHz)	δ(^{13}C) [ppm] (25 MHz)	delta δ(^{13}C) [ppm]	δ(^{13}C) [ppm] (176 MHz)	δ(^{13}C) [ppm] (25 MHz)	delta δ(13C) [ppm]
C=CH	Terminal unit	139.0	139.8	-0.8	139.0	139.9	-0.9
	Inner unit	136.5	137.4	-0.9			
C=CH	Inner unit	126.3	126.9	-0.6			
	Terminal unit	123.8	124.4	-0.6	123.8	124.5	-0.7
C-1	Pseudodisaccharide moiety 4 α-1,4(Glc)	99.9	100.9	-1.0	99.9	100.8	-0.9
		99.6	100.5	-0.9	99.6	100.5	-0.9
		99.5	100.4	-0.9	99.5	100.4	-0.9
		97.5	98.5	-1			
	α,α-1,1 terminal (Glc) inner	93.4	94.2	-0.8	93.4	94.2	-0.8
		93.2	94.0	-0.8	93.2	94.0	-0.8
		77.1	78.0	-0.9	77.0	78.0	-1.0
		69.6	70.4	-0.8	69.7	70.4	-0.7
	Terminal unit	64.9	65.8	-0.9	64.9	65.7	-0.8
	Inner unit	64.2	65.0	-0.8			
	Inner unit	62.0	62.8	-0.8			
	Terminal unit	61.6	62.4	-0.8	61.6	62.5	-0.9
C-6 (Glc)		60.4	61.4	-1.0	60.5	61.4	-0.9
	Terminal unit	56.0	56.8	-0.8	56.0	56.8	-0.8
	Inner unit	55.0	55.9	-0.9	55.0	55.9	-0.9
CH_3-		17.4	18.2	-0.8	17.3	18.2	-0.9

Supplementary Table 4: Molecules Seq2Saccharide failed to predict

Compound	BGC	Failure Mode
avilamycin	BGC0000026	more than five monomers
hygromycin	BGC0000698	large number of genes
apramycin	BGC0000692	mispredicted modifications
fortimicin	BGC0000695	mispredicted modifications
gentamicin	BGC0000696	mispredicted modifications
istamycin	BGC0000700	mispredicted modifications
butirosin	BGC0000693	mispredicted reaction site
sisomicin	BGC0000714	mispredicted reaction site
